# Tuning Reactivity in Cu/TEMPO Catalyzed Alcohol Oxidation Reactions

**DOI:** 10.1002/asia.202500123

**Published:** 2025-04-30

**Authors:** Maximilian Schütze, Matthias Jux, Beatrice Cula, Michael Haumann, Sagie Katz, Peter Hildebrandt, Holger Dau, Kallol Ray

**Affiliations:** ^1^ Institut für Chemie Humboldt‐Universität zu Berlin Brook‐Taylor‐Straße 2 Berlin 12489 Germany; ^2^ Fachbereich Physik Freie Universität Berlin Arnimallee 14 Berlin 14195 Germany; ^3^ Institut für Chemie Fakultät II Technische Universität Berlin Straße des 17. Juni 135 Berlin 10623 Germany

**Keywords:** Aerobic alcohol oxidation, Bioinorganic chemistry, Copper(I) complex, Oxygen activation, Spectroscopy

## Abstract

A dinuclear copper(I) complex **Cu_2_L2_2_** (**L2 **= 3,3‐dimethyl‐1‐(1‐methyl‐1*H*‐benzo[*d*]imidazole‐2‐yl)‐*N*‐(propan‐2‐ylidene)butan‐2‐amine) containing benzimidazole and imino donors was previously reported by some of us as an efficient catalyst for the aerobic oxidation of alcohols to aldehydes in presence of TEMPO (2,2,6,6‐tetramethylpiperidinyloxyl) and an external base NMI (*N*‐methyl imidazole). Cu(III)_2_(bis‐*μ*‐oxo) and Cu(II)_2_(bis‐*μ*‐hydroxo) cores were trapped as viable intermediates in the reaction, which provided deeper mechanistic insights. Here, we report two new ligand systems **L3** (*N*‐isopropyl‐3,3‐dimethyl‐1‐(1‐methyl‐1*H*‐benzol[*d*]imidazole‐2‐yl)butane‐2‐amine) and **L4** ((*Z*)‐2,4‐di‐*tert*‐butyl‐6‐(((3,3‐dimethyl‐1‐(1‐methyl‐1*H*‐benzol[*d*]imidazole‐2‐yl)butane‐2‐yl)imino)methyl)phenol), which are designed to perturb the overall electronics of the complexes and the resulting effects on their O_2_ activation mechanisms. The stronger donation of the secondary amine group stabilizes a mononuclear **Cu^I^L3** core, which nevertheless follows a dinuclear O_2_ activation mechanism as in **Cu_2_L2_2_**. Notably, the **Cu^I^L3**/TEMPO catalyst system performs the aerobic oxidation of alcohols to aldehydes with good yields and turnover numbers, even in the absence of NMI. The dinuclear **Cu^I^_2_L4_2_** complex involving a non‐innocent phenolate group, in contrast, exhibits depleted catalytic activity, because of the instability of the Cu(III)_2_(bis‐*μ*‐oxo) core against intramolecular H‐atom abstraction to form an alkoxo bridged dicopper(II) complex.

## Graphical abstract

Novel copper(I) complexes bearing a secondary amine moiety (**CuL3**) or an additional non‐innocent functionality with intact imine group (**Cu_2_L4_2_**) were synthesized and tested towards oxidation reactions. Several intermediates, such as Cu(III)_2_(bis‐*μ*‐oxo) and Cu(II)_2_(bis‐*μ*‐hydroxo), species were trapped and spectroscopically characterized. Notably, **CuL3** displayed good catalytic activity without the use of an external base whereas the reactivity of **Cu_2_L4_2_** decreased due to a favored self‐oxidation process.

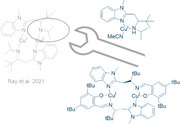

## 1 Introduction

Lytic Polysaccharide Monooxygenase (LPMO)^[^ ^1a–f^ ^]^ and particulate methane monooxygenase (pMMO) ^[^ ^2a–e^ ^]^ are copper‐dependent enzymes with high potential in the field of catalytic degradation of stable compounds. Both enzymes exhibit a very rare histidine‐brace motif for the coordination of the active coppers.^[^ ^3a–d^ ^]^ The type of copper–oxygen intermediates formed during catalysis in pMMO and LPMO is, however, unknown, but they must be highly reactive in order to cleave the very strong C─H bonds of polysaccharides and methane.^[^ ^4a–c^ ^]^ Investigating the mechanisms and structures of biological enzymes can be challenging due to the sensitivity of the protein scaffold, solvent medium, and the instability of reactive intermediates, which complicates spectroscopic studies. Biomimetic complexes that replicate the active centers of enzymes offer a way to gain mechanistic insights, as these synthetic complexes are often stable in organic solvents, allowing structural investigation at low temperatures. As a result, various bioinspired and biomimetic complexes have been synthesized and studied.^[^ ^5a–f^ ^]^

**Scheme 1:**
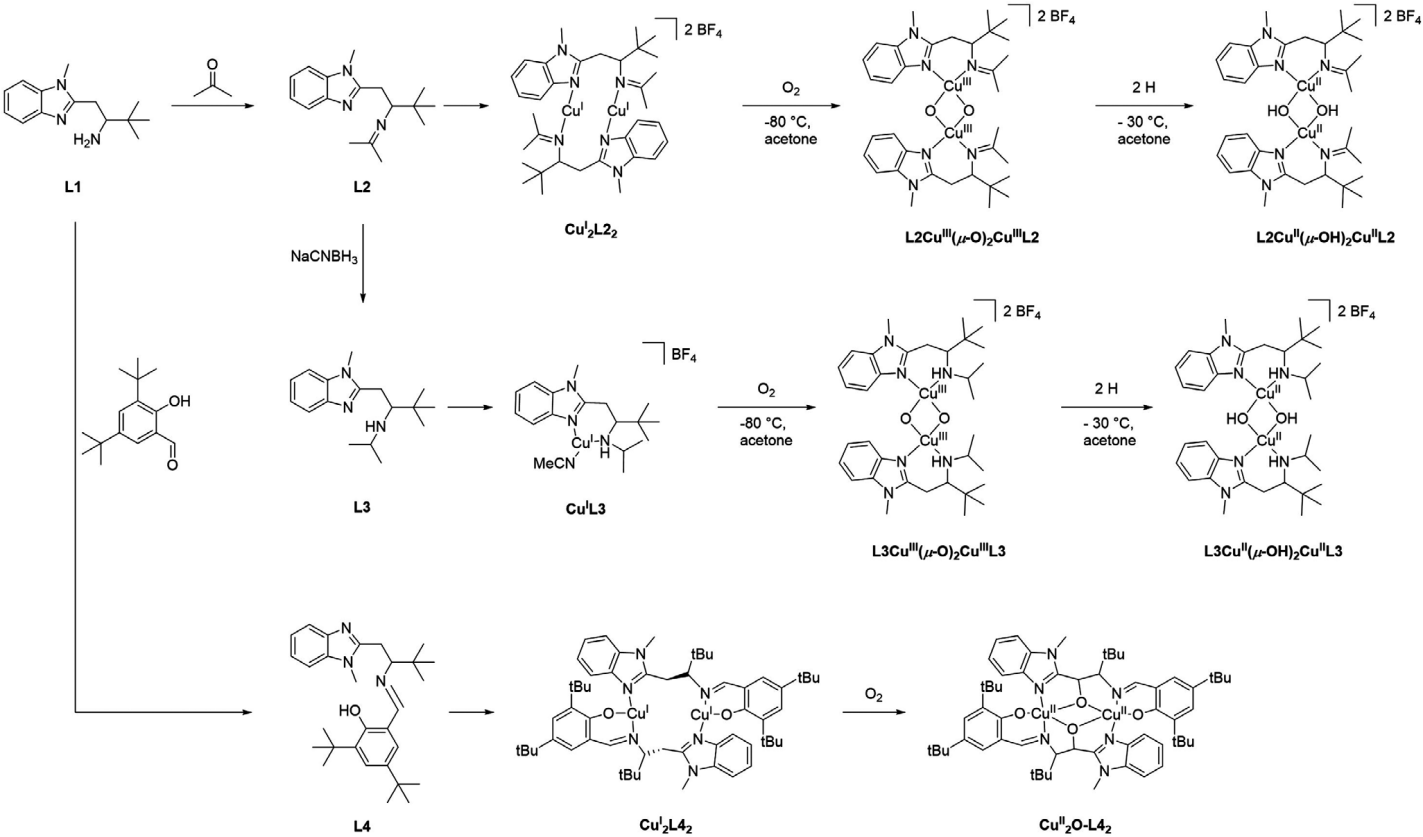
Overview of the synthetic route of **L2**‐**L4** and their corresponding copper complexes. The synthetic route and spectroscopic data for **L1** and **L2** are given in Figures S1–S12.

In our effort on understanding the catalytic mechanism of these copper containing enzymes, we previously reported the synthesis of a “Histidine Brace” ligand (Scheme 1; **L1**) involving a benzimidazole functionality.^[^ ^6^ ^]^ Notably, the primary amine coordination motif of **L1** proved to be too flexible to support binding to a Cu(I) center. Accordingly, no stable [Cu(I)**L1**] complexes could be isolated, which suggests that secondary interaction may be necessary to stabilize the observed coordination of primary amines to Cu(I) centers in biological systems. Subsequent condensation of the ─NH_2_ group of **L1** with acetone led to the synthesis of a modified ligand **L2** containing benzimidazole and imine functionalities. A dinuclear Cu^I^ complex based on **L2** was shown to activate O_2_ to form bis(*μ*‐oxo)dicopper(III) and bis(*μ*‐hydroxo)dicopper(II) species, which could efficiently couple O_2_ reduction and the oxidation of alcohols in presence of TEMPO (TEMPO = 2,2,6,6‐tetramethylpiperidinyloxyl) to act as a suitable catalyst system for the aerobic oxidation of alcohols to aldehydes.^[^ ^5^ ^]^ We could also trap various reactive intermediates involved in the catalytic cycle, which helped us to obtain detailed mechanistic insights which were so far not studied in detail.^[^ ^7a–e^ ^]^

**Scheme 2:**
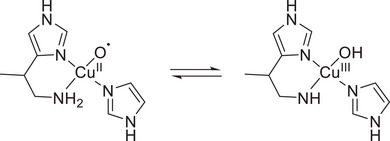
Proposed tautomerization in LPMO.^[^ ^8a^ ^]^

In pMMO or LPMO many proposals for the involvement of mononuclear [Cu^III^‐O]^+^ reactive intermediates have appeared, although such species have never been directly observed.^[^ ^8a–c^ ^]^ By using electron donating‐ligands, Tolman and coworkers^[^ ^8b^ ^]^ have prepared Cu^III^‐OH species, which were found to be strong oxidants. Their reactivity in hydrogen atom abstraction reactions was found significantly higher than that of the most reported metal‐oxo or metal‐hydroxo complexes.^[^ ^8b^ ^]^ Interestingly, the authors have proposed that such Cu^III^‐OH species could be generated in the active site of LPMO upon protonation of a [Cu^III^‐O]^+^ species via tautomerization involving the primary amine of the histidine‐brace (Scheme 2).^[^ ^8a^ ^]^ Deprotonation of the primary amine could then provide the necessary electronic donation in order to get appropriate basicity and reactivity of the [CuOH]^+^ core.

The proposed tautomerization and amine deprotonation can also take place in secondary amines. Accordingly, in the present study we have performed reductive amination of **L2** with NaCNBH_3_ to generate a new ligand **L3** containing benzimidazole/imidazole and secondary amine functionalities. We presumed that the Cu^III^(*μ*‐O)_2_Cu^III^ intermediate formed upon O_2_ activation can yield a mononuclear Cu^III^‐OH moiety in an equilibrium step stabilized by tautomerization in presence of a secondary amine. The generated Cu^III^‐OH species can then perform alkane oxidation reactions, in contrast to the observed selectivity of the Cu^II^‐OH species towards alcohol oxidation.^[^ ^8b^ ^]^ We also extended the coupling chemistry of ligand **L1** to include an aldehyde containing a redox non‐innocent phenolate ligand to form a ligand **L4**. We anticipated that the relative stabilities of the various intermediates formed upon O_2_ activation can be altered by the presence of ligand non‐innocence, which will affect the product selectivity and also the nature of the substrate (alcohol vs. alkane oxidation reactions).

## 2 Results and Discussion

### 2.1 Synthesis and Characterization of the Ligands and Their Corresponding Cu(I) Complexes

**Ligand Synthesis**: The synthesis of **L3** was conducted by mild reduction of the imine group in **L2** using NaCNBH_3_ in anhydrous methanol (Scheme S1). The formation of **L3** was confirmed by ^1^H‐NMR spectroscopy. Peaks at *δ*  =  0.82 and 0.41 ppm as doublets, as well as a new chemical shift at *δ*  =  2.17 ppm appeared as a septet confirming the reduction of the iso‐propylidene to an iso‐propyl group (Figure S13). In ESI‐MS measurements, a signal at m/z  = 274.2 could be observed matching the calculated value of m/z = 274.2 for the fragment [M + H]^+^, therefore confirming the formation of the product (Figure S14). In infrared (IR) spectroscopic measurements, a new signal appeared at *ṽ* = 3320 cm^−1^, which can be assigned to a N─H stretch motion of a secondary amine (Figure S15). The purity of the new compound was confirmed by elemental analysis (EA). For single crystal X‐ray diffraction (sc‐XRD) measurements, single crystals were obtained by hot recrystallization from ethyl acetate. The ligand **L3** crystallizes in the space group P 21/C. A bond distance between N_amine_ and C_amine_ of 1.472(1) Å can be observed. As the bond distance increased in **L3** compared to the imine precursor **L2** (1.281(3) Å), the presence of a secondary amine could be assumed. The obtained bond distance is also in agreement with the literature known C─N_amine_ bond distances (Figure S16).

The ligand (*Z*)‐2,4‐di‐*tert*‐butyl‐6‐(((3,3‐dimethyl‐1‐(1‐methyl‐1*H*‐benzol[*d*]imidazole‐2‐yl)butane‐2‐yl)imino)methyl)phenol (**L4**), containing a phenol moiety, was synthesized in three‐steps (Scheme S1). The reductive amination step was performed analogous to a reported synthesis route of Itoh et al.^[^ ^3c^ ^]^ Hereby, 3,5‐di‐*tert*‐butyl‐2‐hydroxybenzaldehyde was used as a coupling reagent for 3,3‐dimethyl‐1‐(1‐methyl‐1*H*‐benzol[*d*]imidazole‐2‐yl)butane‐2‐amine. **L4** was analyzed by various analytical methods. In the ^1^H‐NMR spectrum, new peaks at *δ* = 14.01 and 8.18 ppm occurred, which can be assigned to the hydrogen atoms of the hydroxyl group and the C═N moiety, respectively. The signals are shifted down‐field relative to **L2**, which is expected due to the electron‐withdrawing nature of the phenolate moiety. Additional peaks in the aromatic region could be observed. Moreover, two high‐field shifted signals at *δ* = 1.41 and 1.19 ppm appearing as singlets can be seen, which can be assigned to the hydrogen atoms of the newly incorporated di‐*tert*‐butyl groups (Figure S17). ESI‐MS measurements of **L4** were performed. Signals at m/z = 448.4, 470.3, 486.3, and 511.4 can be observed matching the calculated values of m/z = 448.3, 470.3, 486.3, and 511.3 of the ions [M + H]^+^, [M + Na]^+^, [M + K]^+^, and [M + CH_3_CN + Na]^+^, respectively, confirming the successful formation of **L4** (Figure S18). In the IR spectrum, new signals at 2834 and 1361 cm^−1^ can be observed, which can be assigned to O─H stretching and O─H bending modes, respectively, confirming the presence of the incorporated di‐*tert*‐butyl phenol moiety (Figure S19). EA analysis confirmed the purity of the ligand **L4**. Structural insights were gained by sc‐XRD measurements of crystals grown by hot recrystallization from ethyl acetate. The ligand **L4** crystallizes in the space group P 21/n. The bond distance between N_imine_ and C_imine_ is 1.287(5) Å, thus confirming the presence of an imine moiety, due to the short bond length as observed in 3,3‐dimethyl‐1‐(1‐methyl‐1*H*‐benzol[*d*]imidazole‐2‐yl)‐*N*‐propane‐2–3‐ylidene)butane‐2‐amine (Figure S20).

**Figure 1:**
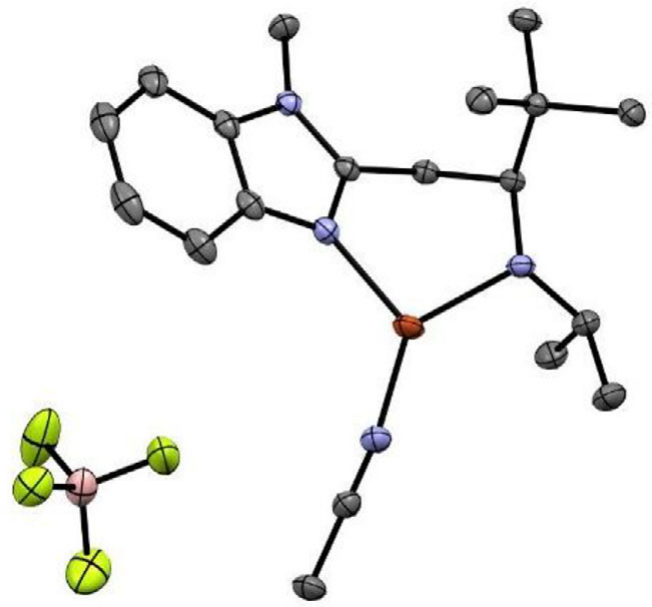
Molecular structure of mononuclear **[** **Cu^I^L3]BF_4_** as obtained from sc‐XRD measurements. The structures are shown in ORTEP representation with thermal ellipsoids at 50% probability level. H atoms are omitted for clarity. (Color code: Cu: orange; N: blue, C: grey; B: pink; F: yellow.)^[^ ^9^ ^]^

**Copper complexes**: Metalation of **L3** with tetrakis(acetonitrile)copper(I) tetrafluoroborate led to the formation of **Cu^I^L3** in 55% yield (Schemes 1 and S1). ESI‐MS measurements of **Cu^I^L3** exhibited prominent signals at m/z = 609.4 and 377.1, matching the calculated values of m/z 609.4 and 377.2 for the ion fragments [Cu + 2**L1**]^+^ and [Cu + **L1 **+ MeCN]^+^, respectively (Figure S21). **Cu^I^L3** is diamagnetic (S = 0), as confirmed by ^1^H‐NMR (Figure S22) spectroscopy. The chemical shifts range from 0 to +10 ppm and are shifted relative to the free ligand signals confirming the binding of **L3** to copper. X‐band EPR spectroscopic measurements revealed only 0.6 % (*g*_⊥_ = 2.06 and *g_∥_*  = 2.23) of a minor EPR active species compared to a copper(II) standard, suggesting the main species to be EPR silent (Figure S23). Structural insights of **Cu^I^L3** were gained by sc‐XRD measurements. Crystals were grown by layering diethyl ether to a saturated solution of **Cu^I^L3** in MeCN and cooled down to −40 °C. The molecular structure is shown in Figure 1. **Cu^I^L3** adopts a mononuclear, trigonal‐planar geometry with a non‐binding BF_4_^−^ counter ion, which is in sharp contrast to the dinuclear copper imine complex **Cu^I^_2_L2_2_**. The bond distance between N_amine_ and C_amine_ is 1.507(3) Å, confirming the presence of the secondary amine moiety in **Cu^I^L3**. The bond distances of N_amine_, N_bzim_ and N_MeCN_ to the Cu center are 2.059(2), 1.990(2), and 1.880(2) Å, respectively, displaying a longer metal‐ligand distance compared to **Cu^I^_2_L2_2_** (Table 1).

**Table 1:** Selected bond distances of the complexes **Cu** ^**I**^_**2**_ **L2**_**2**_,^[^ ^5^ ^]^ **Cu** ^**I**^ **L3**, **Cu** ^**I**^_**2**_ **L4**_**2**_, and their oxygen intermediates.

Bond Distance (Å)	Cu^I^_2_L2_2_	L2Cu^III^(*μ*‐O)_2_Cu^III^L2^a)^	L2Cu^II^(*μ*‐OH)_2_Cu^II^L2^a)^	Cu^I^L3	L3Cu^III^(*μ*‐O)_2_Cu^III^L3^a)^	L3Cu^II^(*μ*‐OH)_2_Cu^II^L3^a)^	Cu^I^_2_L4_2_	Cu^II^_2_O‐L4_2_
Cu–Cu	3.144(1) 3.19^a)^	2.76	3.01	–	2.83	2.99	3.452(5)	3.177(1)
Cu–N_bzim_	1.877(4) 1.91^a)^	1.96	1.96	1.990(2) 1.94^a)^	1.97	1.99	1.911(1)/1.921(1)	2.022(3)
Cu–N_amine/imine_	1.903(4) 1.91^a)^	1.96	1.96	2.059(2) 1.94^a)^	1.97	1.99	1.906(1)/1.906(1)	1.915(5)
Cu–N_MeCN_	–	–	–	1.880(2)	–	–	–	–
Cu–O_phen_	–	–	–	–	–	–	2.060(1)/2.110(1)	1.909(4)
Cu–O	–	1.81	1.96	–	1.97	1.99	–	1.948(4)

^a)^ (mean) interatomic distances determined by EXAFS analysis (see Table S1).

**Figure 2:**
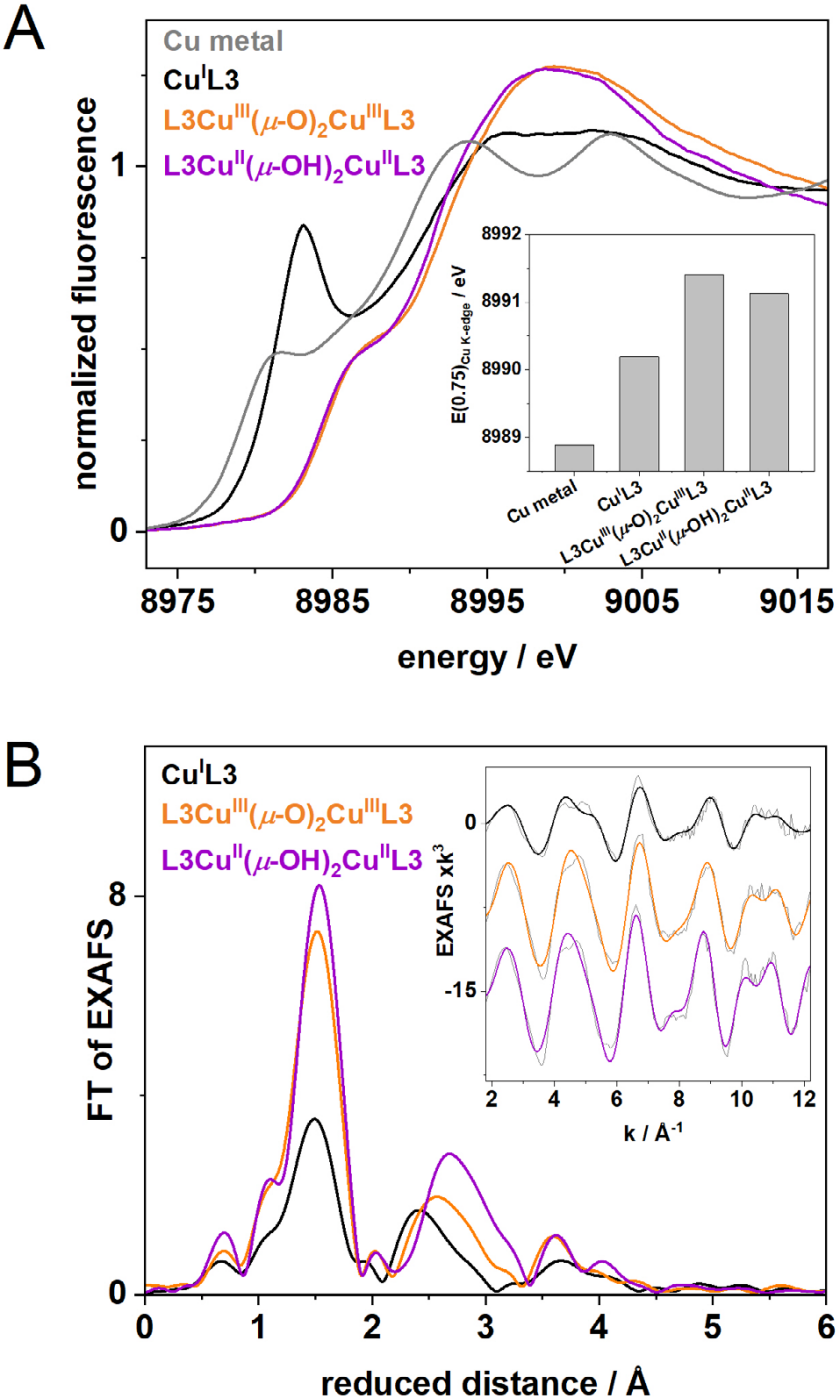
Cu XAS spectra. A) XANES spectra of **Cu^I^L3**, **L3Cu^III^(*μ*‐O)_2_Cu^III^L3**, **L3Cu^II^(*μ*‐OH)_2_Cu^II^L3,** and a Cu metal foil reference. Cu K‐edge energies at 0.75 level are shown in the inset. B) Fourier‐transforms of EXAFS spectra in the inset (stacked for clarity; black lines, experimental data; colored lines, simulations).

X‐ray absorption near‐edge structure (XANES) spectra at the Cu‐K‐edge for **Cu^I^L3** showed the typical peak feature around 8983 eV (when compared, e.g., to the Cu metal spectrum) in the K‐edge rise, which is indicative of Cu(I). Extended X‐ray absorption fine structure (EXAFS) analysis revealed ca. 2 N/O ligands with a mean bond length of about 1.95 Å (Tables 1 and S1) which is slightly shorter compared to the crystal structure (∼2.03 Å), possibly reflecting increased covalency of the Cu─N bonds in solution. In addition, EXAFS analysis suggested the absence of the MeCN molecule of solid **Cu^I^L3** when the complex was dissolved in acetone. No significant copper‐copper interaction was detectable by EXAFS, supporting the fact that the complex retains its mononuclear structure in solution (Figure 2).

**Figure 3:**
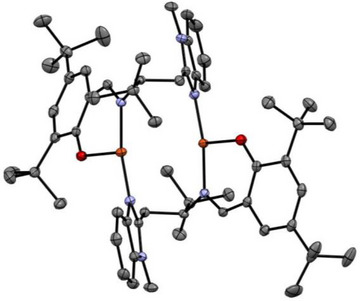
Molecular structure of dinuclear **Cu** ^**I**^_**2**_ **L4**_**2**_ as obtained from sc‐XRD measurements. The structures are shown in ORTEP representation with thermal ellipsoids at 50% probability level. H atoms are omitted for clarity. (Color code: Cu: orange; N: blue, O: red, C: grey.)^[^ ^9^ ^]^

When **L4** was metalated with Cu(I) acetate a bright yellow solid **Cu^I^_2_L4_2_** is formed in quantitative yield (Schemes 1 and S1). The ESI‐MS spectrum of **Cu^I^_2_L4_2_** exhibits a prominent signal at m/z = 551.2, matching the calculated value of m/z 551.3 for the ion fragment [Cu + **L4 **+ MeCN + H]^+^ (Figure S24). In the ^1^H‐NMR spectrum of **Cu^I^_2_L4_2_**, a shift of the signals compared to the signals of free ligand in the range of 0–10 ppm can be observed, confirming the presence of a diamagnetic Cu(I) species (Figure S25). Only 5.2% (*g*_⊥_ = 2.05 and *g_∥_* = 2.34) of an EPR active species were detected (Figure S26). Crystals suitable for sc‐XRD measurements were grown via inert, hot recrystallization in benzene. **Cu^I^_2_L4_2_** is a neutral complex and exhibits a dinuclear structure. Each Cu ion is coordinated in a trigonal‐planar geometry. The bond distances of N_imine_, N_bzim_, and O_phen_ to the two Cu centers are 1.906(1)/1.906(1), 1.911(1)/1.921(1), and 2.060(1)/2.110(1) Å, respectively. The Cu─N bond distances are similar to the reported ones of **Cu^I^_2_L2_2_** proving the Cu(I) assignment. Also, the Cu─O_phen_ bond distances are comparable to the ones found in mononuclear Cu(I)‐phenolate complexes.^[^ ^10a,b^ ^]^ Interestingly, the Cu─Cu distance in **Cu^I^_2_L4_2_** is 3.453(5) Å, which is remarkably longer than in the previously reported dinuclear Cu complex **Cu^I^_2_L2_2_** (3.144(1) Å). Notably, **Cu^I^_2_L4_2_** represents a rare example of a dinuclear Cu(I) species with a non‐bridging phenolate ligand (Figure 3).

### 2.2 Dioxygen Activation Reactions

**Figure 4:**
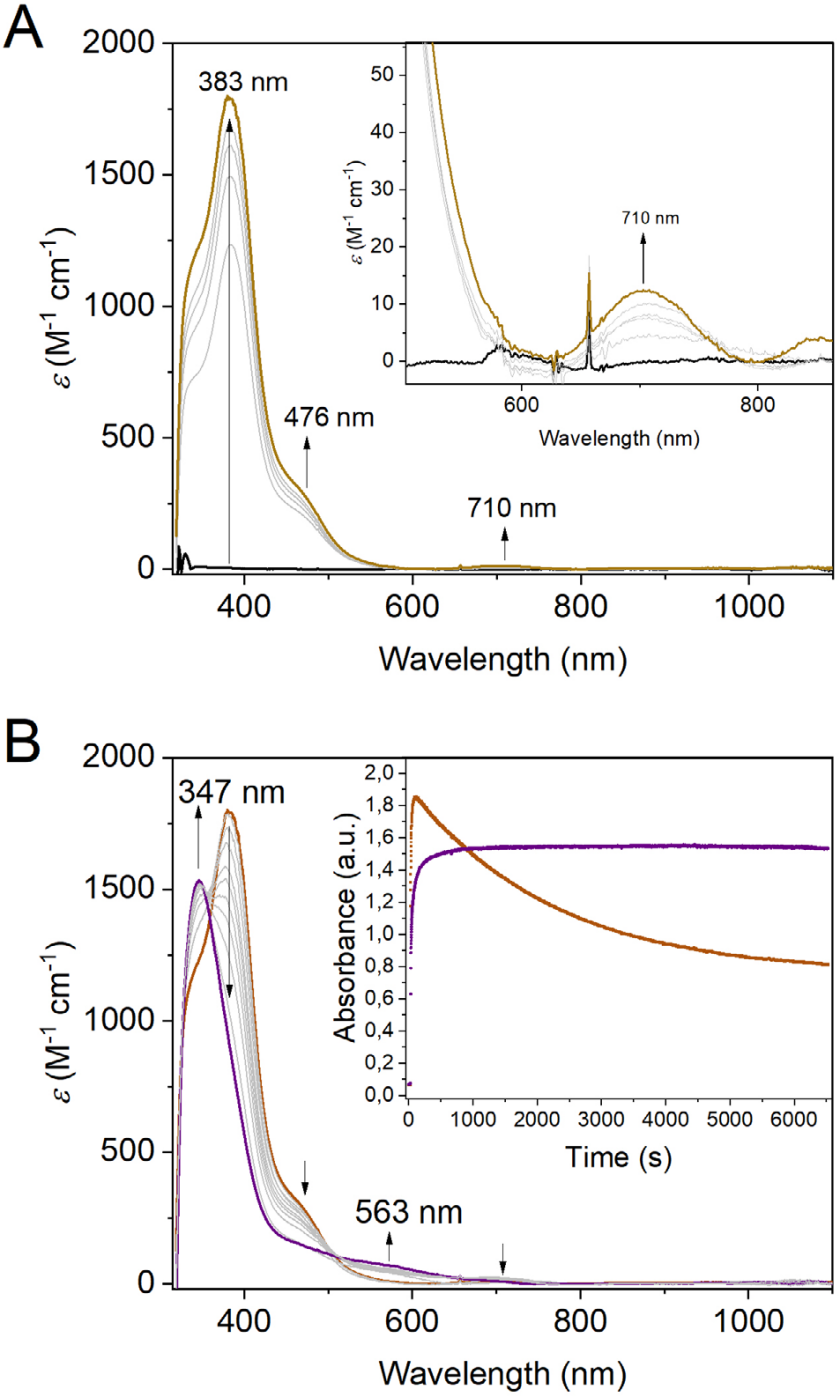
A) UV/Vis spectra of the formation of **L3Cu** ^**III**^ **(*μ*‐O)**_**2**_ **Cu** ^**III**^ **L3** (yellow trace) during the reaction of **Cu** ^**I**^ **L3** (black trace, 1 mM in acetone) with O_2_ at −90 °C; in the inset is given the expansion of the 500‐850 nm region; B) Conversion of **L3Cu** ^**III**^ **(** ***μ*‐O** **)**_**2**_ **Cu** ^**III**^ **L3** (yellow trace) to **L3Cu** ^**II**^ **(** ***μ*‐OH** **)**_**2**_ **Cu** ^**II**^ **L3** (violet trace) in acetone at −90 °C. The inset shows the time trace of the bands at 383 nm (yellow trace) and 347 nm (violet trace).

**Figure 5:**
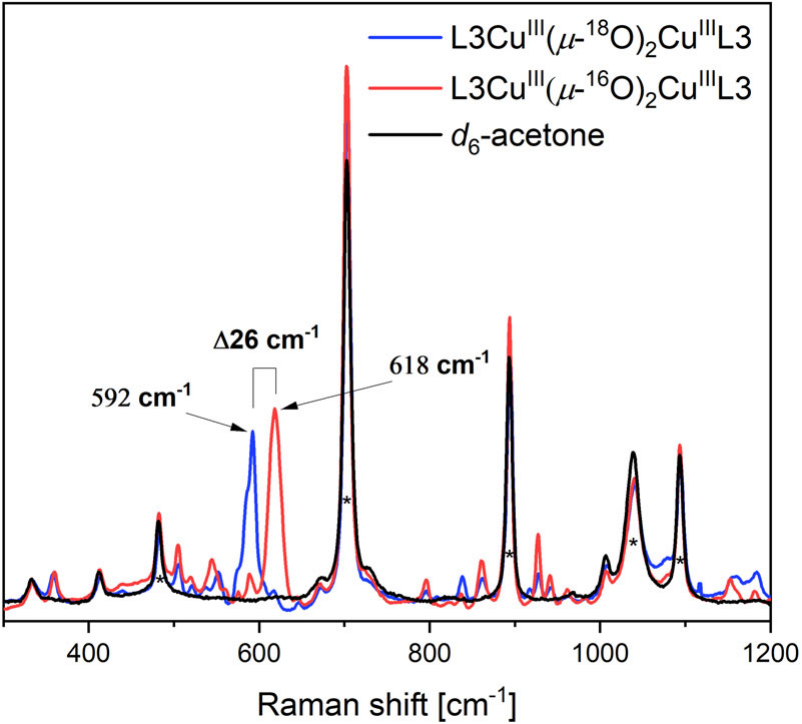
rRaman spectra of ^16^O‐labelled **L3Cu^III^(*μ*‐^16^O)_2_Cu^III^L3** (red) and ^18^O‐labelled **L3Cu^III^(*μ*‐^18^O)_2_Cu^III^L3** (blue) upon 406 nm Kr^+^ laser irradiation at −95 °C. Conc: 10 mM in d_6_‐acetone.

**Reaction of Cu^I^L3**: Interestingly, in spite of its mononuclear nature, the O_2_ reactivity of **Cu^I^L3** is found to be comparable to that of **Cu^I^_2_L2_2_;** it reacts with O₂ in acetone at −90 °C to yield a new, transient species (*t*_1/2_ ≈ 24 min at −80 °C in *d*_*6*_‐acetone), which displays absorption maxima at 383 nm (*ε*_max_ = 1850 M^−1^ cm^−1^), 476 nm (*ε*_max_ = 320 M^−1^ cm^−1^) and 714 nm (*ε*_max_ = 70 M^−1^ cm^−1^) (Figure 4a). rRaman measurements revealed an oxygen‐sensitive band at 618 cm^−1^, which shifts to 592 cm^−1^ upon ^18^O‐labelling (Figure 5). This shift (^16/18^Δ = 26 cm^−1^) is consistent with the formation of dinuclear **L3Cu^III^(*μ*‐O)_2_Cu^III^L3** upon activation of O_2_ at a mononuclear **Cu^I^L3** center as described in the literature.^[^ ^11a–c^ ^]^ The Cu K‐edge XANES spectrum of **L3Cu^III^(*μ*‐O)_2_Cu^III^L3** showed an edge energy about 1.5 eV higher than the one of **Cu^I^L3**, presumably indicating a mixture of (mostly) Cu(II) species with a smaller contribution of Cu(III) species, due to incomplete complex oxidation because of the higher sample concentration (5 mM) in the XAS experiments. The mixture of Cu(II)/Cu(III) species was also apparent in the EXAFS data, which revealed a large increase of the number of first‐sphere ligands, i.e., 4–5 N/O ligands with a mean bond length of about 1.97 Å, being slightly longer compared to the bond lengths reported for **L2Cu^II^(*μ*‐OH)_2_Cu^II^L2**. Additionally, two sub‐stoichiometric Cu─Cu distances close to ca. 2.85 and 3.0 Å are compatible with the EXAFS data, supporting the presence of dinuclear structures. We tentatively attribute the shorter distance with a smaller coordination number to the Cu^III^(bis‐*μ*‐O)Cu^III^ dimer and the longer distance with a slightly larger coordination number to the Cu^II^(bis‐*μ*‐OH)Cu^II^ dimer (see below and Figure 2, Tables 1 and S1).

**L3Cu^III^(*μ*‐O)_2_Cu^III^L3** reacted with substrates containing C─H bonds like 1,4‐CHD, fluorene and dihydroanthracene (DHA) as evident from the decay of its characteristic UV features at 383 and 710 nm in presence of these substrates forming **L3Cu^II^(*μ*‐OH)_2_Cu^II^L3** (see below). The second‐order rate constant (*k*_2_) of the reaction with 1,4‐CHD has been determined to be 0.097 M^−1^ s^−1^ at −90 °C (Figure S27), which is comparable to the value reported previously for **L3Cu^III^(*μ*‐O)_2_Cu^III^L3**. Product analysis has been done for the reaction with DHA. Gas‐chromatography flame ionization detection (GC‐FID) analysis revealed the formation of anthraquinone in 16% yield relative to the complex concentration (Figure S28).

**L3Cu^III^(*μ*‐O)_2_Cu^III^L3** is EPR silent (95.2% yield; Figure S23) and converts at higher temperature to another EPR silent species (97.9% yield; Figure S23), which is assigned to **L3Cu^II^(*μ*‐OH)_2_Cu^II^L3** based on its absorption features at 347 nm (*ε*_max_ = 1550 M^−1^ cm^−1^), and 563 nm (*ε*_max_ = 140 M^−1^ cm^−1^) (Figure 4b). The Cu XANES of **L3Cu^II^(*μ*‐OH)_2_Cu^II^L3** shows an edge‐energy ca. 0.5 eV lower than that **L3Cu^II^(*μ*‐OH)_2_Cu^II^L3**, consistent with merely Cu(II) in the former sample. EXAFS analysis revealed close to 4 Cu‐N/O scatterers at 1.99 Å, that is, slightly longer than for the Cu^III^ dimer (see above). Again, two sub‐stochiometric Cu─Cu distances of close to 2.85 and 3.0 Å are compatible with the EXAFS data. While the coordination number of the shorter distance was negligible, due to the mere absence of this species in the sample, the N‐value of the longer distance was larger than for the Cu^III^ containing sample, supporting the attribution of the longer distance to the Cu^II^(bis‐*μ*‐OH)Cu^II^ dimer (Tables 1 and S1). Both the metal‐ligand bond length and the Cu─Cu distance are comparable to the previously reported value for **L2Cu^II^(*μ*‐OH)_2_Cu^II^L2** ^[^ ^3^ ^]^ (Table 1). The proposed dinuclear, hydroxo‐bridged diamond‐core structure in **L3Cu^II^(*μ*‐OH)_2_Cu^II^L3** is also verified by sc‐XRD measurements (Figure S29); however, any detailed discussion on metrical parameters is precluded because of the insufficient quality of the crystal structure.

**L3Cu^II^(*μ*‐OH)_2_Cu^II^L3** also performed HAA from dihydroanthracene to form anthracene in 24% yield. Two additional products, phorone and 4‐hydroxy‐4‐methylpentane in average yields of 18% and 51%, respectively, were detected (Figure S30). These are products presumably associated with HAA from acetone during the conversion of **L3Cu^III^(*μ*‐O)_2_Cu^III^L3** to **L3Cu^II^(*μ*‐OH)_2_Cu^II^L3**.

**Figure 6:**
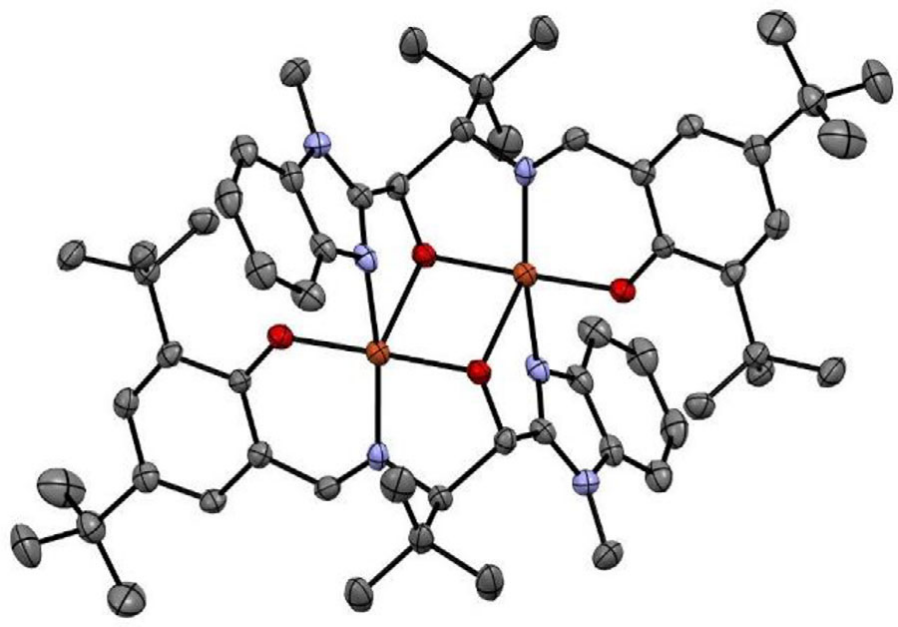
Molecular structure of one independent molecule in the asymmetric unit of **Cu** ^**II**^_**2**_ **O** **‐** **L4**_**2**_ × 1 C_2_H_3_N × 1 C_7_H_8_O obtained from sc‐XRD measurements. The structure is shown in ORTEP representation with thermal ellipsoids at 50% probability level. H atoms and solvent molecules are omitted for clarity. (Color code: Cu: orange; N: blue, O: red, C: grey.)^[^ ^9^ ^]^

**Reaction of Cu^I^_2_L4_2_**: The presence of additional non‐innocent phenolate groups in **Cu^I^_2_L4_2_** alters its O_2_ reactivity significantly relative to **Cu^I^L3** and **Cu^I^_2_L2_2_**. In presence of O_2_ **Cu^I^_2_L4_2_** decays spontaneously to an alkoxo‐bridged dicopper(II) species **Cu^II^_2_O‐L4_2_**, whose structure is shown in Figure 6. Each Cu ion is coordinated in a distorted square‐pyramidal geometry displaying a diamond‐core structure. Although the metal‐ligand bond distances are comparable to **Cu^I^_2_L4_2_**, the Cu─Cu distance is much shortened (3.117(1) Å) due to the binding of the alkoxo groups. The Cu─O bond distance is 1.948(4) Å which is significantly shortened relative to **Cu^I^_2_L4_2_** and comparable to the Cu^II^─O distances observed in **L2Cu^II^(*μ*‐OH)_2_Cu^II^L2** (1.96 Å) and **L3Cu^II^(*μ*‐OH)_2_Cu^II^L3** (1.93 Å), which may suggest a Cu(II) assignment.

No intermediates were detected during the decay of **Cu^I^_2_L4_2_** (Figure S31). Thus, the HAA ability of the initially formed **L4Cu^III^(*μ*‐O)_2_Cu^III^L4** intermediate is presumably increased in presence of a non‐innocent phenolate group, so that intramolecular HAA occurs very fast giving rise to the alkoxo‐bridged product.

### 2.3 Catalytic Oxidations of Alcohols

Both **Cu^I^L3** and **Cu^I^_2_L4_2_** were tested for their ability to perform aerobic oxidation of benzyl alcohol under catalytic conditions (Equation 1).

(1) $$O_{2}+2{\mathrm{PhCH}}_{2}\mathrm{OH}\underset{\begin{array}{c} \mathrm{actone}\\ 20^{\circ }C,8h \end{array}}{\overset{\begin{array}{c} \left[\mathrm{Cu}\right]\left(4\mathrm{mol}\%\right),\\ \mathrm{TEMPO}\left(2\mathrm{mol}\%\right), \end{array}}{\to }}2H_{2}O+2\mathrm{PhCHO}$$

While **Cu^I^_2_L4_2_** is catalytically inactive in presence of O_2_ or air in acetone at 20 °C, **Cu^I^L3** (4 mol %) and TEMPO (2 mol %) can catalytically oxidize benzyl alcohol to benzaldehyde with an excellent yield of 69% even in the absence of NMI. The catalytic performance of **Cu^I^L3** is therefore also better than **Cu^I^_2_L2_2_**, which yielded benzaldehyde in only 20% yield under similar conditions (Table S10). As suggested in our previous study, NMI acts as a base to assist the formation of the Cu^II^‐alcoholate complex and to facilitate the dissociation of the **[** **L2** **Cu** ^**II**^ **(*μ*‐OH)**_**2**_ **Cu** ^**II**^ **L2** **]** ^**2+**^ dimer into the more reactive **[** **L1** **Cu(OH)]** ^**+**^ monomer. **Cu^I^L3** includes a secondary ─NH group in the ligand backbone, which can function as a base, and also facilitate the monomeric structure, because of its stronger donating properties; hence **Cu^I^L3** is catalytically efficient even in the absence of NMI. This is also evident in the case of more challenging aliphatic substrates like *n*‐hexanol. Whereas hexanal is obtained in only 2% yield for the **Cu^I^_2_L2_2_**/O_2_/TEMPO catalytic system, a significantly higher yield (18%) of the aldehyde product is observed for **Cu^I^L3**/O_2_/TEMPO (Table S10).

## 3 Conclusion

**Scheme 3:**
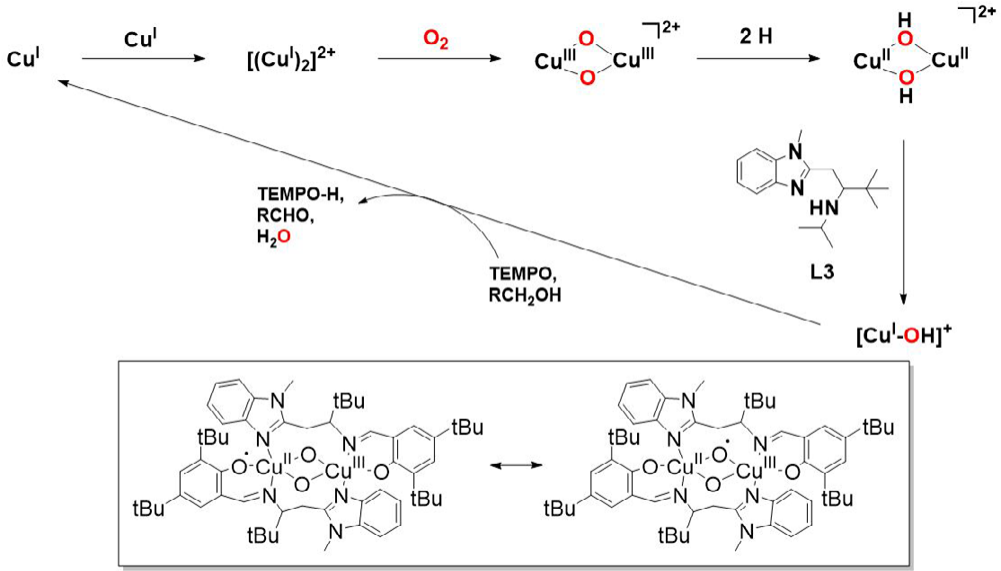
Proposed catalytic cycle for the base‐free aerobic alcohol oxidation by **Cu** ^**I**^ **L3**. Inset: Mesomeric structures of a possible **L4Cu** ^**III**^ **(*μ*‐O)**_**2**_ **Cu** ^**III**^ **L4** intermediate leading to fast self‐oxidation to **Cu** ^**II**^_**2**_ **O‐L4**_**2**_.

In summary, similar to the previously reported **Cu^I^_2_L2_2_**/O_2_/TEMPO/NMI catalytic system, bis(*μ*‐oxo)dicopper(III) and bis(*μ*‐hydroxo)dicopper(II) species have been identified and spectroscopically characterized as reactive intermediates in **Cu^I^L3**/TEMPO catalyzed aerobic oxidation of benzyl alcohol and *n*‐hexanol. The presence of a secondary ─NH group in **L3** presumably facilitates the generation of the monomeric Cu^II^‐OH reactive intermediate from the bis(*μ*‐hydroxo)dicopper(II) species (Scheme 3), which allows the system to be active even in the absence of NMI. In contrast, **Cu^I^_2_L4_2_** is catalytically inactive; in presence of oxygen, it favors a fast self‐oxidation reaction which leads to the incorporation of oxygen into the ligand backbone to form an alkoxo‐bridged species, which hampers further substrate oxidation reactions. The presence of a non‐innocent phenolate ligand in **L4** presumably introduces sufficient oxyl radical character in the initially formed bis(*μ*‐oxo)dicopper(III) species by valence delocalization, which makes it unstable against the self‐decomposition process. The protection of the ‐CH_2_ group might terminate the self‐oxidation process and facilitate external C─H bond activation reactions, which will be targeted in future studies.

## Supporting Information

The authors have included additional spectroscopic and crystallographic data as well as cited references within the Supporting Information.^[^ ^12^, ^13^, ^14^, ^15^, ^16^, ^17^, ^18^ ^]^

## Conflict of Interests

The authors declare no conflict of interest.

## Supporting information



Supporting Information

## Data Availability

The data that support the findings of this study are available from the corresponding author upon reasonable request.
