# Supplementary material for: Tuning Reactivity in Cu/TEMPO Catalyzed Alcohol Oxidation Reactions
Source: Chem Asian J. 2025 Apr 30;20(11):e202500123. doi: 10.1002/asia.202500123 (PMC12182405; doi:10.1002/asia.202500123)
Supplement: Supplementary file 1 — Supporting Information [file ASIA-20-e202500123-s001.pdf]

Supporting Information

Tuning Reactivity in Cu/TEMPO Catalyzed Alcohol Oxidation Reactions

*Maximilian Schütze, Matthias Jux, Beatrice Cula, Michael Haumann, Sagie Katz, Peter Hildebrandt, Holger Dau, and Kallol Ray**

Table of Contents

Table of Contents.....2

Experimental Procedures.....2

Materials and Instrumentations.....2

Synthesis Procedures.....3

Generation of the intermediates.....5

Sample preparation and catalytic studies.....6

Results and Discussion.....7

Experimental Procedures

Materials and Instrumentations

Chemicals and handling. All chemicals used were purchased from the companies ABCR, SIGMA-ALDRICH, TCI, and BLDpharm and used without further purification unless required. Anhydrous solvents (acetonitrile, diethyl ether, dichloromethane and acetone) were purchased from CARL-ROTH GmbH under the tradename ROTIDRY (>99.5%, <50 ppm H_2O). Deuterated solvents were purchased from EURISO-TOP. Methanol for synthesis purposes was dried using a literature known procedure: To 100 mL of distilled methanol was added 0.5 g of iodine-activated magnesium. After reflux for two hours, 350 mL of distilled methanol was added and the reaction mixture was distilled under inert conditions into a Young flask. Then, the freshly distilled methanol was purged with argon for 10 min, 90 mg of activated 3 Å molecular sieve was added and the flask was kept in the fridge for five days prior use.

Preparation and handling of air or water sensitive compounds were performed under an inert atmosphere using either Schlenk techniques or a MBraun glovebox filled with N_2. Nitrogen and argon of quality 5.0 were used for this purpose and were purchased from AIR LIQUIDE.

Elemental analysis. All elemental analyses were performed by the analytical service of the Institut für Chemie und Biochemie of the Freie Universität Berlin. The percentages of Carbon, Hydrogen, Nitrogen and Sulphur were determined using a VARIO EL analyzer. The reported values for the ligands are the result of an average of two independent measurements.

Nuclear magnetic resonance spectroscopy. All NMR spectra were recorded using a BRUKER AVANCE DPX 300 spectrometer or a BRUKER AVANCE III 500 spectrometer. ^{1}H and ^{13}C nuclei were recorded in deuterated solvents, and signals (ppm) referenced against residual protic solvent peaks.

Electrospray ionization mass spectrometry. ESI-MS spectra of organic molecules and inorganic complexes in solution were recorded by using an ADVION EXPRESSION CMS spectrometer (in positive ionization mode); acetonitrile or methanol were used as eluent. The analysis of the data was carried out with the ADVION DATA EXPRESS Version 6.0.11.3.

Gas chromatography. GC-FID analysis was carried out by using an AGILENT 7890B gas chromatograph (HP5 column, 30 m) with a flame-ionization detector. GC-MS was performed on an AGILENT 5977B spectrometer with a triple-axis detector.

Single crystal X-ray diffraction. Data collection was performed at 100 K on a BRUKER D8 VENTURE diffractometer by using Mo Kα = radiation (λ = 0.71073 Å). Multi-scan absorption correction implemented in SADABS[12] was applied to the data. The structure was solved by intrinsic phasing method (SHELXT 2014/5)[13] and refined by full-matrix-least-square procedures based on F^2 with all measured reflections (SHELXL-2018/3)[14] in the graphical user interface (SHELXle)[15] with anisotropic temperature factors for all non-hydrogen atoms. All hydrogen atoms were added geometrically and refined by using a riding model. Crystallographic data are displayed in Table S2-S9. Deposition numbers 2411973 (for 3-amino-4,4-dimethylpentanoic acid × H_2O), 2411974 (for **L1**), 2411975 (for **L2**), 2411976 (for **L3**), 2411977 (for **L4**), 2388744 (for **Cu^{I}L3**), 2411978 (for **Cu^{I}_2L4$_2$**), 2411979 (for **Cu^{II}_2O-L4$_2$** × 2 $C_2H_3N)_2$ and 2411980 (for Cu^{II}_2O-L4$_2$ × C_2H_3N × $C_7H_8O)_2$) contain the supplementary crystallographic data for this paper. These data are provided free of charge by the joint Cambridge Crystallographic Data Centre and Fachinformationszentrum Karlsruhe Access Structures service.[9]

Electron paramagnetic resonance spectroscopy. X-band EPR spectra were collected by a Bruker EMXplus Instrument. All samples were measured as frozen solutions by the use of the liquid helium recirculating cooling system provided by ColdEdge. Data handling and quantification of the signals were performed by comparing the area of the double-integrated experimental signals with a copper(II) standard (1 mM $CuSO_4$ in H_2O) and by the use of easyspin software.[16] Typical

sample concentration was 1 mM in either acetone or acetonitrile (temperature: 13 K, modulation amplitude: 5 G, power: 0.02518 mW, 39 dB). The experimental spectra were baseline corrected.

X-ray absorption spectroscopy. XAS at the Cu K-edge was performed at beamline KMC-3 at the BESSY-II synchrotron (Helmholtz Center Berlin, Germany) using a set-up including a Si[111] double-crystal monochromator, a 13-element energy-resolving Si-drift detector (RaySpec) for X-ray fluorescence monitoring, and DXP-XMAP pulse-processing electronics (XIA). Samples were held at 20 K in a liquid-helium cryostat (Oxford). The energy axis of the monochromator was calibrated (accuracy ±0.1 eV) using the K-edge spectrum of a copper metal foil (fitted reference energy of 8979 eV in the first derivative spectrum). The spot size on the samples was ca. 1.5 x 3.0 mm (vertical x horizontal) as set by a focusing mirror and slits. X-ray fluorescence spectra were collected using a continuous scan mode of the monochromator (scan duration ~10 min). Up to 6 scans were averaged (1-2 scans per sample spot) for signal-to-noise ratio improvement. XAS data were processed (dead-time correction, background subtraction, normalization) to yield XANES and EXAFS spectra using our earlier described procedures and in-house software.[17] k^3-weighted EXAFS spectra were simulated with in-house software and phase functions from FEFF9 (S_0^2 = 1.0).[18] The simple geometric model for calculation of phase functions contained 6 1st-sphere Cu-N/O distances in a 1.8-2.3 Å range, 3 Cu-C distances in a 2.8-3.5 Å range, and 2 Cu-Cu distances of 3.0 Å and 3.3 Å. Mean 1st sphere bond lengths were calculated by the following formula: $=N_iR_i/=N_i$. EXAFS simulation results are tabulated in Table S1.

Cyclic voltammetry. The CV experiments were performed with a CH-Instrument potentiostat. Acetone for **Cu^{I}L3** or MeCN for **Cu$^I_2$L4$_2$** were used as solvents and tetrabutylammonium hexafluorophosphate ([(n-Bu)$_4$N]PF$_6$, 0.1 M) as supporting electrolyte. The CV measurements were performed in a three electrode setup using a platinum working electrode, a platinum wire counter electrode and a Ag/AgNO$_3$ pseudo reference electrode under nitrogen atmosphere at 20 °C. The potential was referenced against ferrocene which was measured under identical conditions.

Resonance Raman. Resonance Raman spectra were measured in solution state at −95 °C (Bruker cryostat) with 406 nm excitation (Kr$^+$-laser) using a Horiba Jobin-Yvon LabRAM HR800 confocal Raman spectrometer. The concentrations of samples were around 10 mM. Spectra were obtained at a laser power of 4 mW with an accumulation time of about 10 min.

Synthesis Procedures

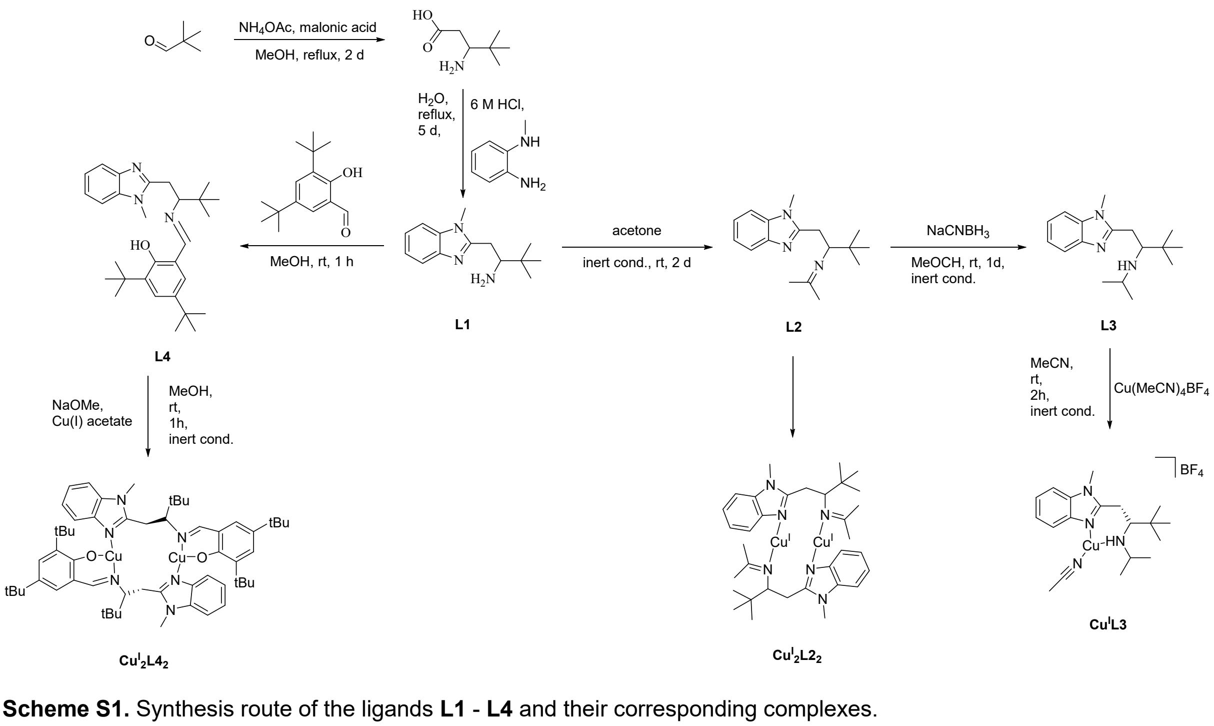

Scheme S1. Synthesis route of the ligands **L1** - **L4** and their corresponding complexes.

Synthesis of 3-amino-4,4-dimethylpentanoic acid. Malonic acid (5.00 g, 48.0 mmol, 1.0 eq), pivalaldehyde (5.38 g, 62.4 mmol, 1.3 eq) and ammonium acetate (5.56 g, 72.0 mmol, 1.5 eq) were suspended in 90 mL of ethanol and stirred under reflux for 2 d. Subsequently, around 90% of the solvent was removed under reduced pressure. A white precipitate was formed after the addition of 100 mL of ethyl acetate to the remaining solution and filtered to yield 3-amino-4,4-dimethylpentanoic acid (3.63 g, 25.1 mmol, 52%) as a white solid.

^{1}H-NMR (500 MHz, D_3O): δ [ppm] = 3.29 (dd, $_{HH}J^3$ = 10.6 Hz, $_{HH}J^2$ = 3.3 Hz, 1H, H_4), 2.61 (dd, $_{HH}J^3$ = 17.1 Hz, $_{HH}J^2$ = 3.4 Hz, 1H, H_4), 2.35 (dd, $_{HH}J^3$ = 17.0 Hz, $_{HH}J^3$ = 10.7 Hz, 1H, H_5), 0.99 (s, 9H, $H_{8,9,10}$).

ESI-MS (pos. mode, m/z): $[M+CH_3CN+H]^+$ calc.: 187.1, exp.: 187.2.

ATR-IR (in cm^{-1}): $\nu_{C–N}$ = 1158, $\nu_{C–N}$ = 1226, $\delta_{C–H}$ = 1374, $\delta_{N–H}$ = 1633, $\nu_{O–H}$ = 3262 - 2551 (centered peak at 2952), $\nu_{N–H}$ = 3377. 51.

Synthesis of 3,3-dimethyl-1-(1-methyl-1*H*-benzol[*d*]imidazole-2-yl)butan-2-amine (L1). *N*-methyl-1,2-phenylenediamine (1.96 mL, 17.2 mmol, 1.0 eq.) and 3-amino-4,4-dimethylpentanoic acid (3.00 g, 20.7 mmol, 1.2 eq) were dissolved in 10 mL of 6 M HCl solution and refluxed for 6 d. Afterwards, the reaction mixture was cooled to 0 °C and a 6 M sodium hydroxide solution was added dropwise to adjust the pH value to >12. A precipitation of a dark solid was observed. The reaction mixture was extracted with methylene chloride (5x 50 mL). The organic phases were collected, dried over MgSO4, filtered and the solvent was removed under reduced pressure to afford a dark solid. The crude product was purified by column chromatography (aluminum oxide, DCM/MeOH 50:1→10:1, *R* = 0.3) to afford a brown solid, which was purified further by extraction with *n*-hexane to afford 3,3-dimethyl-1-(1-methyl-1*H*-benzol[*d*]imidazole-2-yl)butane-2-amine (0.75 g, 3.2 mmol, 18%) as a beige solid.

^{1}H-NMR (500 MHz, $CDCl_3$): δ [ppm] = 7.62 (dd, $_{HH}J^4$ = 7.2 Hz, $_{HH}J^3$ = 1.4 Hz, 1H, H_2), 7.33 (dd, $_{HH}J^4$ = 7.2 Hz, $_{HH}J^3$ = 1.5 Hz, 1H, H_1), 7.27 – 7.16 (m, 2H, $H_{3,6}$), 3.72 (s, 3H, H_{10}), 3.11 – 3.01 (m, 2H, H_{11}), 2.61 (dd, $_{HH}J^3$ = 15.2 Hz, $_{HH}J^3$ = 11.2 Hz, 1H, H_{12}), 1.03 (s, 9H, $H_{15,16,17}$).

ESI-MS (pos. mode, m/z): $[M+H]^+$ calc.: 232.2, exp.: 232.2.

ATR-IR (in cm^{-1}): $\nu_{C–N}$ = 1095, $\nu_{C–N}$ = 1232, $\nu_{C–N}$ = 1328, $\delta_{N–H}$ = 1610, $\nu_{C–H}$ = 2952, $\nu_{O–H}$ = 3052, $\nu_{N–H}$ = 3361.

Synthesis of 3,3-dimethyl-1-(1-methyl-1*H*-benzol[*d*]imidazole-2-yl)-*N*-(propane-2-ylidene)butane-2-amine (L2). Under nitrogen atmosphere, 100 mg of 3,3-dimethyl-1-(1-methyl-1*H*-benzol[*d*]imidazole-2-yl)butane-2-amine were dissolved in 2.5 mL of anhydrous acetone and stirred at room temperature for 48 h. The solvent was removed under reduced pressure and the product was obtained quantitatively as a beige solid.

^{1}H-NMR (500 MHz, $(CD_3)_2CO$): [ppm] = 7.51 (dd, $_{HH}J^4$ = 7.6 Hz, $_{HH}J^3$ = 1.8 Hz, 1H, H_2), 7.37 (dd, $_{HH}J^4$ = 7.2 Hz, $_{HH}J$ = 1.5 Hz, 1H, H_1), 7.20 – 7.09 (m, 2H, $H_{3,6}$), 3.73 (s, 3H, H_{10}), 3.67 (dd, $_{HH}J^3$ = 10.4 Hz, $_{HH}J^2$ = 2.6 Hz, 1H, H_{11}), 3.11 (dd, $_{HH}J^3$ = 13.8 Hz, $_{HH}J^2$ = 2.6 Hz, 1H, H_{11}), 2.95 (m, $_{HH}J^3$ = 13.8 Hz, $_{HH}J^3$ = 10.4 Hz, 1H, H_{12}), 1.75 (s, 3H, H_{19}), 1.24 (s, 3H, H_{20}), 0.99 (s, 9H, $H_{15,16,17}$).

ESI-MS (pos. mode, m/z): $[M+H]^+$ calc.: 272.2, exp.: 272.3.

ATR-IR (in cm^{-1}): $\nu_{C–N}$ = 1329, $\nu_{C=N}$ = 1664, $\nu_{C–H}$ = 2952, $\nu_{O–H}$ = 3055.

Synthesis of *N*-isopropyl-3,3-dimethyl-1-(1-methyl-1H-benzol[*d*]imidazole-2-yl)butane-2-amine (L3). Under argon atmosphere, 3,3-dimethyl-1-(1-methyl-1H-benzol[d]imidazole-2-yl)-N-(propane-2-ylidene)butane-2-amine (100 mg, 0.37 mmol, 1 eq.) was dissolved in 5 mL of dry methanol. Solid sodium cyanoborohydride (51 mg. 0.81 mmol, 2.2 eq.) was added and the reaction mixture was stirred for 2 days. Afterwards, 90% of the solvent was evaporated and 10 mL of an aqueous 6 M NaOH solution was added. The aqueous solution was extracted three times with 15 mL of DCM. The organic phases were combined, dried over MgSO4, filtered and the solvent was removed under reduced pressure to afford *N*-isopropyl-3,3-dimethyl-1-(1-methyl-1*H*-benzol[*d*]imidazole-2-yl)butane-2-amine as a brown solid (81 mg, 0.30 mmol, 80%).

^{1}H-NMR (500 MHz, $(CD_3)_2CO$): δ [ppm] = 7.56 (dd, $_{HH}J^4$ = 7.0, $_{HH}J^3$ = 1.4 Hz, 1H, H_2), 7.42 (dd, $_{HH}J^4$ = 7.1, $_{HH}J^3$ = 1.5 Hz, 1H, H_1), 7.20 – 7.12 (m, 2H, $H_{3,6}$), 3.87 (s, 3H, H_{10}), 3.19 (dd, $_{HH}J^3$ = 13.5, $_{HH}J^2$ = 2.9 Hz, 1H, H_{11a}), 2.78 – 2.66 (m, 2H, $H_{11b,12}$), 2.17 (hept, $_{HH}J^3$ = 6.2 Hz, 1H, H_{18}), 0.82 (d, $_{HH}J^4$ = 6.2 Hz, 3H, H_{19}), 0.41 (d, $_{HH}J^4$ = 6.2 Hz, 3H, H_{20}).

ESI-MS (pos. mode, m/z): $[M+H]^+$ calc.: 274.2, exp.: 274.2.

ATR-IR (in cm^{-1}): $\nu_{C–N}$ = 1334, $\nu_{C–H}$ = 2952, $\nu_{O–H}$ = 3050, $\nu_{N–H}$ = 3320.

EA (in %): exp.: C: 77.74, H: 9.05, N: 8.85, calc.: C: 77.36, H: 9.23, N: 9.21 for $C_{39}H_{41}N_3O$ x 0.1 $C_4H_8O_2$.

Synthesis of (*Z*)-2,4-di-*tert*-butyl-6-(((3,3-dimethyl-1-(1-methyl-1*H*-benzol[*d*]imidazole-2-yl)butane-2-yl)imino)methyl)phenol (L4). Under argon atmosphere, solid Na (87.5 mg, 3.8 mmol, 2.2 eq.) was dissolved in 10 mL of anhydrous MeOH and stirred at room temperature for 15 min. Afterwards, 3,3-dimethyl-1-(1-methyl-1*H*-benzol[*d*]imidazole-2-yl)butane-2-amine (400.0 mg, 1.7 mmol, 1 eq.) was added as solid and the yellow reaction mixture was stirred at room temperature for 30 min. Solid 3,5-di-*tert*-butylphenol-1-benzaldehyde (405.2 mg, 1.7 mmol, 1 eq.) was added at room temperature and the reaction mixture turned bright yellow. After stirring for an hour, the solvent was removed under reduced pressure. To the remaining solid, an aqueous 7.5 mM NaOH solution was added and extracted with methylene chloride (3x 30 mL). The organic phase was dried over $MgSO_4$, and the solvent was removed under reduced pressure to afford a yellow solid. The crude product was recrystallized from hot ethyl acetate to afford 3,3-dimetyhl-1-(1-methyl-1*H*-benzol[*d*]imidazole-2-yl)-*N*-(2,4-di-*tert*-butylphenol-6-ylidene)butane-2-amine as yellow crystals (753 mg, 1.6 mmol, 97%).

^{1}H-NMR (500 MHz, $(CD_3)_2CO$): δ [ppm] = 14.01 (s, 1H, H_{24}), 8.18 (s, 1H, H_{17}), 7.57 – 7.50 (m, 1H, H_2), 7.34 (d, $_{HH}J^3$ = 2.5 Hz, 1H, H_1), 7.31 – 7.25 (m, 1H, H_{23}), 7.15 – 7.08 (m, 2H, $H_{3,6}$), 6.93 (m, 1H, H_{21}), 3.72 (s, 3H, H_{33}), 3.69 (dd, $_{HH}J^3$ = 10.2, $_{HH}J^2$ = 2.5 Hz, 1H, H_{10a}), 3.36 (dd, $_{HH}J^3$ = 15.2, $_{HH}J^2$ = 2.5 Hz, 1H, H_{10b}), 3.24 (dd, $_{HH}J^3$ = 15.2, $_{HH}J^3$ = 10.2 Hz, 1H, H_{11}), 1.41 (s, 9H, $H_{26,27,28}$), 1.19 (s, 9H, $H_{30,31,32}$), 1.12 (s, 9H, $H_{13,14,15}$).

ESI-MS (pos. mode, m/z): $[M+H]^+$ calc.: 448.3, exp.: 448.4, $[M+Na]^+$ calc.: 470.3, exp.: 470.3, $[M+K]^+$ calc.: 486.3, exp.: 486.3, $[M+CH_3CN+Na]^+$ calc.: 511.3, exp.: 511.4.

ATR-IR (in cm^{-1}): δ_{O-H} = 1361, ν_{O-H} = 2834, ν_{C-H} = 2952, ν_{O-H} = 3055.

EA (in %): exp.: C: 77.74, H: 9.05, N: 8.85, calc.: C: 77.36, H: 9.23, N: 9.21 for $C_{39}H_{41}N_3O$ x 0.1 $C_4H_8O_2$.

Synthesis of Copper(I) [*N*-isopropyl-3,3-dimethyl-1-(1-methyl-1*H*-benzol[*d*]imidazole-2-yl)butane-2-amine] tetrafluoroborate (Cu^{I}L3). Under nitrogen atmosphere, *N*-isopropyl-3,3-dimethyl-1-(1-methyl-1H-benzol[*d*]imidazole-2-yl)butane-2-amine (50 mg, 0.18 mmol, 1.1 eq.) was dissolved in 2 mL of anhydrous methylene chloride. Solid tetrakis(acetonitrile)copper(I) tetrafluoroborate (52 mg, 0.17 mmol, 1 eq.) was added and the brown solution was stirred overnight. Afterwards, 15 mL of anhydrous diethyl ether were added, and the precipitation of a white solid could be observed. The reaction mixture was cooled to –40 °C overnight for complete precipitation. The solution was decanted off and the remaining solid was washed with 2 mL of anhydrous diethyl ether. The solid was dried under reduced pressure to obtain Copper(I) [*N*-isopropyl-3,3-dimethyl-1-(1-methyl-1*H*-benzol[*d*]imidazole-2-yl)butane-2-amine] tetrafluoroborate as an off-white powder (25 mg, 0.05 mmol, 32%).

ESI-MS (pos. mode): [Cu+**L1**]$^+$ calc.: 337.2, exp.: 377.1, [Cu+2**L1**]$^+$ calc.: 609.4, exp.: 609.4.

EA (in %): exp.: C: 51.01, H: 8.66, N: 12.78, calc.: C: 51.99, H: 7.59, N: 11.77 for $CuC_{29}H_{30}N_4BF_4$ x 1.15 $C_4H_{10}O$ x 0.95 CH_3CN.

Synthesis of dicopper(I) bis[(*Z*)-2,4-di-*tert*-butyl-6-(((3,3-dimethyl-1-(1-methyl-1*H*-benzol[*d*]imidazole-2-yl)butane-2-yl)imino)methyl)phenolate] (Cu$^I_2$L4$_2$). Under nitrogen atmosphere, solid sodium (23 mg, 1.00 mmol, 1.5 eq.) was dissolved in 5 mL of anhydrous MeOH and stirred at room temperature for 15 min. Afterwards, (*Z*)-2,4-di-*tert*-butyl-6-(((3,3-dimethyl-1-(1-methyl-1*H*-benzol[*d*]imidazole-2-yl)butane-2-yl)imino)methyl)phenol (300 mg, 0.67 mmol, 1.1 eq) was added as solid and the yellow reaction mixture was stirred at room temperature for 30 min. Solid copper(I) acetate (78 mg, 0.64 mmol, 1 eq.) was added at room temperature and the reaction mixture turned orange. The formation of a precipitate could be observed. After stirring for an hour, the reaction mixture was filtered off and the solid was washed twice with 2 mL of anhydrous MeOH to obtain dicopper(I) bis[(*Z*)-2,4-di-*tert*-butyl-6-(((3,3-dimethyl-1-(1-methyl-1*H*-benzol[*d*]imidazole-2-yl)butane-2-yl)imino)methyl)phenolate] as bright yellow powder (290 mg, 0.57 mmol, 89%).

ESI-MS (pos. mode): [Cu+**L2**+MeCN+H]$^+$ calc.: 551.3, exp.: 551.2.

EA (in %): exp.: C: 67.15, H: 7.64, N: 7.54, calc.: C: 66.86, H: 7.93, N: 7.84 for $CuC_{39}H_{40}N_3O$ x 0.3 $C_4H_{10}O_2$ x 0.25 CH_3OH.

Generation of the intermediates

Formation of intermediate L3Cu^{III}(μ-O)$_2$Cu^{III}L3. In a typical experiment, a solution of **Cu^{I}L3** in deuterated acetone was prepared and filled in the cuvette. After cooling down to –90 °C, molecular oxygen was bubbled through the solution for ten seconds. The immediate formation of the characteristic bands of **L3Cu^{III}(μ-O)$_2$Cu^{III}L3** could be observed.

Formation of intermediate L3Cu^{II}(μ-OH)$_2$Cu^{II}L3. In a typical experiment, a solution of **Cu^{I}L3** in acetone was prepared and filled in the cuvette. After cooling down to –30 °C, molecular oxygen was bubbled through the solution for ten seconds. The immediate formation of the characteristic bands of **L3Cu^{II}(μ-OH)$_2$Cu^{II}L3** could be observed.

Sample preparation and catalytic studies

General procedure for reactivity studies and GC sample preparation. In a typical experiment, a 0.5 mM solution of the intermediates were formed with molecular oxygen at −90 °C (**L3Cu^{III}(μ-O)$_2$Cu^{III}L3**) or −10 °C (**L3Cu^{II}(μ-OH)$_2$Cu^{II}L3**) (cuvette pathlength: 0.5 cm, cuvette volume: 1 mL). Afterwards, argon was bubbled through the intermediate solution to ensure the absence of excess oxygen. Then, the substrate (10 eq. to 40 eq.) dissolved in 0.05 mL anhydrous and degassed solvent was added with a syringe. The decay of the bands at λ = 383 nm and 714 nm (**L3Cu^{III}(μ-O)$_2$Cu^{III}L3**) and 347 nm (**L3Cu^{II}(μ-OH)$_2$Cu^{II}L3b**) were observed. For GC product analysis, a known amount of biphenyl standard in ethyl acetate was added to a solution of 0.25 mM intermediate with 10 eq. substrate. The solution was filtered through a short plug silica column and washed with an additional 0.5 mL ethyl acetate.

Determination of second-order rate constants (*k*$_2$). Reactivity studies were performed on solutions of (**L3Cu^{III}(μ-O)$_2$Cu^{III}L3**) at –90 °C with >2.5 mM concentrations. After removing excess oxygen (by bubbling Ar through the solution for 2 min), a solution of the substrates (>20 eq) was added to a solution of the preformed intermediate and the decay of its respective UV-Vis features (714 nm) was monitored acquiring an UV-vis spectrum every second fitted with pseudo-first order kinetics to obtain k_{obs} values which were found to be linearly increasing with the increase of the substrate concentrations. The second order rate constant (k_2) values were provided by the slope of the rate constant k_{obs} vs substrate concentration plot.

General procedure for catalytic aerobic alcohol oxidation with external base. Catalytic alcohol oxidation reactions were performed similar to the reported procedure of *Ray et al.* In a typical experiment, solutions containing 0.01 mmol of the respective Cu(I) catalysts (10 mol% of **Cu^{I}L3** in d6-acetone or 5 mol% of **Cu$^{I}_2$L4$_2$** in d3-acetonitrile), 0.01 mmol TEMPO radical (5 mol%) and 0.02 mmol *N*-methyl imidazole (10 mol%) were prepared in a total volume of 1.25 mL using anhydrous solvents inside of a glovebox. The solutions were taken out of the glovebox and opened to air. Afterwards, 0.2 mmol of the respective alcohol were added to the solution and the reaction mixture was stirred at room temperature for 3 h. After this time, 0.02 mmol of 1,3,5-trimethoxybenzene was added to the solutions and the solutions were filtered through a short plug silica column to remove the catalyst. The product was washed down from the silica plug with 0.5 mL of the respective solvent. The yields of the aldehyde products were quantified by the ratio of the ^{1}H-NMR signals of the aldehyde CHO signal vs. the signals of 1,3,5-trimethoxybenzene.

General procedure for catalytic aerobic alcohol oxidation without external base. Catalytic alcohol oxidation reactions without the use of NMI were performed similar to the reported procedure of *Ray et al. I*. In a typical experiment, solutions containing 0.004 mmol of the respective Cu(I) catalysts (4 mol% of **Cu^{I}L3** in d6-acetone or 2 mol% of Cu$^{I}_2$L4$_2$ in d3-acetonitrile, 0.002 mmol TEMPO radical (2 mol%) were prepared in a total volume of 1.25 mL using anhydrous solvents inside of a glovebox. The solutions were taken out of the glovebox and opened to air. Afterwards, 0.1 mmol of the respective alcohol were added to the solution and the reaction mixture was stirred at room temperature for 3 h. After this time, 0.01 mmol of 1,3,5-trimethoxybenzene was added to the solutions and the solutions were filtered through a short plug silica column to remove the catalyst. The product was washed down from the silica plug with 0.5 mL of the respective deuterated solvent. The yields of the aldehyde products were quantified by the ratio of the ^{1}H-NMR signals of the aldehyde CHO signal vs. the signals of 1,3,5-trimethoxybenzene.

Results and Discussion

Table S1. EXAFS simulation parameters for **$Cu^{I}L3$**, **$L3Cu^{III}(\mu\text{-}O)_2Cu^{III}L3$** and **$L3Cu^{II}(\mu\text{-}OH)_2Cu^{II}L3$** (interatomic distance, R [Å]; coordination number, N [per Cu]; Debye-Waller factor, $2\sigma^2$ x10^3 [Å^2]; fit error sum, R_F(1-3.5 Å) [%]).

**shell**		Cu–N/O	Cu–N/O	Cu–C	Cu–C	Cu–Cu	Cu–Cu	R_F
**$Cu^{I}L3$**	R	1.89	2.29	2.91	3.34	-	-	17.4
	N	1.8	0.3	3*	1*	-	-	
	$2\sigma^2$	7#	7#	8#	8#	-	-	
**$L3Cu^{III}(\mu\text{-}O)_2Cu^{III}L3$**	R	1.91	2.35	2.94	3.33	2.83	2.97	11.8
	N	3.9	0.6	3*	1*	0.2	0.3	
	$2\sigma^2$	8#	8#	10#	10#	2*	2*	
**$L3Cu^{II}(\mu\text{-}OH)_2Cu^{II}L3$**	R	1.94	2.37	2.96	3.35	2.85	2.99	10.1
	N	4.1	0.5	3*	1*	<0.1	0.6	
	$2\sigma^2$	7#	7#	10#	10#	2*	2*	

#, Debye-Waller parameters ($2\sigma^2$ x10^3 [Å^2]) that were coupled to yield the same values for more than one shell in the EXAFS fits; *, fixed parameters.

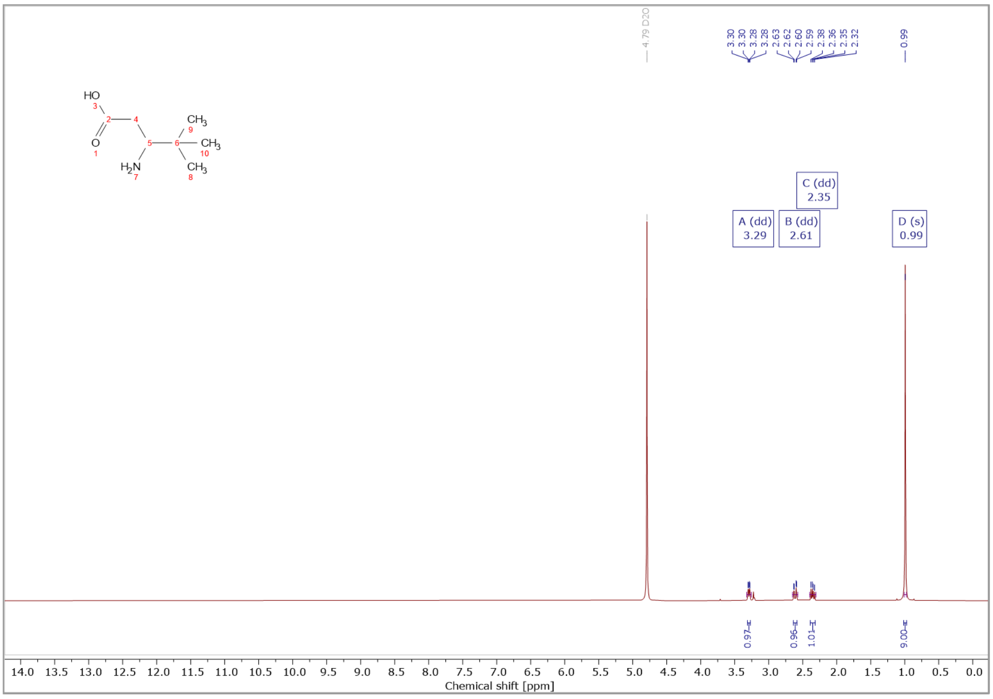

Figure S1. ^{1}H-NMR spectrum of 3-amino-4,4-dimethylpentanoic acid in D_2O at 20 °C.

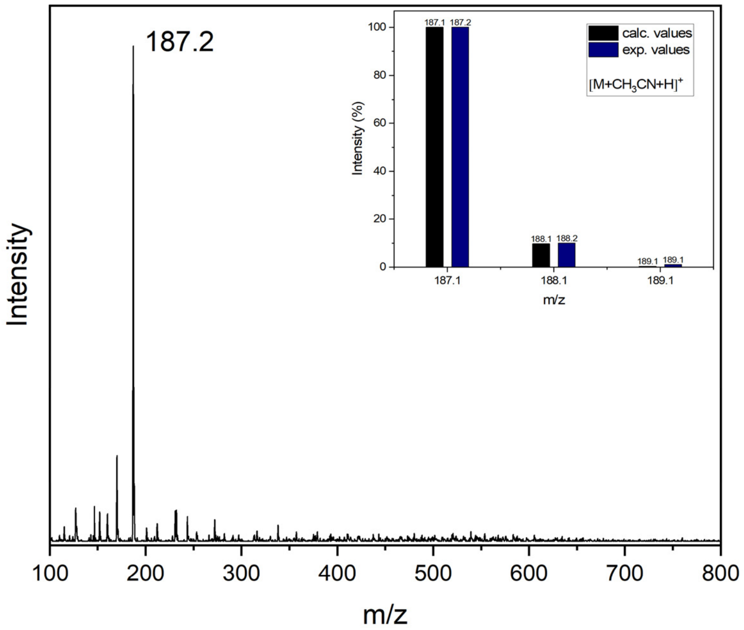

Figure S2. ESI-MS spectrum (positive mode) of 3-amino-4,4-dimethylpentanoic acid with comparison of the experimental obtained data (blue) and calculated data (black).

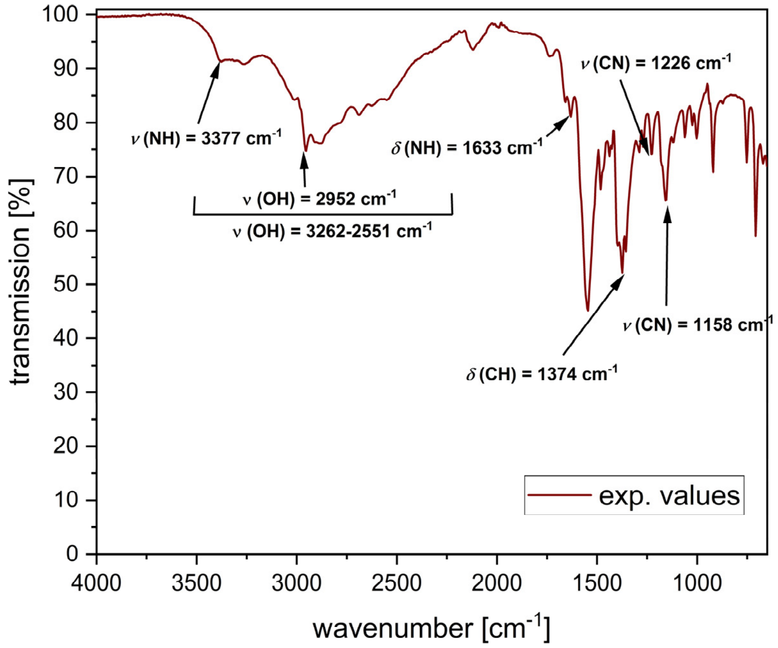

Figure S3. ATR-IR spectrum of 3-amino-4,4-dimethylpentanoic acid at 20 °C.

13

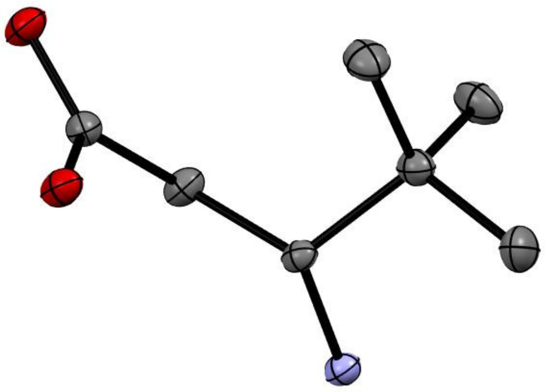

Figure S4. Molecular structure of 3-amino-4,4-dimethylpentanoic acid × H_2O obtained from sc-XRD measurements. The structure is shown in ORTEP representation with thermal ellipsoids at 50% probability level. H atoms and solvent molecules are omitted for clarity. (Color code: N: blue, O: red, C: grey.)

Table S2. Crystallographic data of 3-amino-4,4-dimethylpentanoic acid × H_2O obtained from sc-XRD measurements.

Chemical sum formula	$C_7H_{17}NO_3$
Chemical moiety formula	$C_7H_{15}NO_2$ × H_2O
Molecular weight	163.21 g/mol
Temperature	100(2) K
Wavelength	0.71073 Å
Crystal system	monoclinic
Space group	*P*2/*c*
Unit cell parameters	*a* = 13.4047(12) Å
	b = 5.9716(5) Å
	c = 11.3737(8) Å
	α = 90 °
	β = 91.975(4) °
	γ = 90 °
Cell volume	V = 909.90(13) Å3
Z	4
Crystal density	1.191
Absorption coefficient *μ*	0.092
F(000)	360
Crystal size	0.720 x 0.180 x 0.050 mm
Θ_{min} to θ_{max}	3.412 ° to 28.293 °
h, k, l range	-17 ≤ h ≤ 17; -7 ≤ k ≤ 7; -14 ≤ l ≤ 15
Total number of reflections (with I > 2σ(I))	2239
Number of parameters/restraints	123/0
R_1 factor	0.0386
wR_2 factor	0.0957
Goodness of Fit (GooF)	1.022
Deposition number	2411973

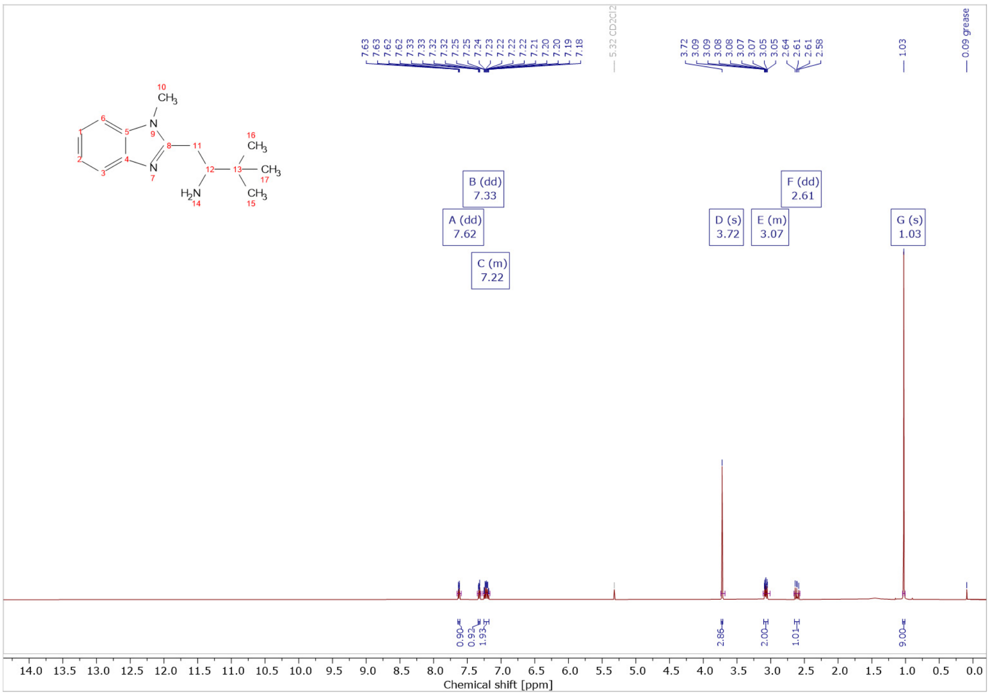

Figure S5. ^{1}H-NMR spectrum of 3,3-dimethyl-1-(1-methyl-1*H*-benzol[*d*]imidazole-2-yl)butane-2-amine (**L1**) in $CDCl_3$ at 20 °C.

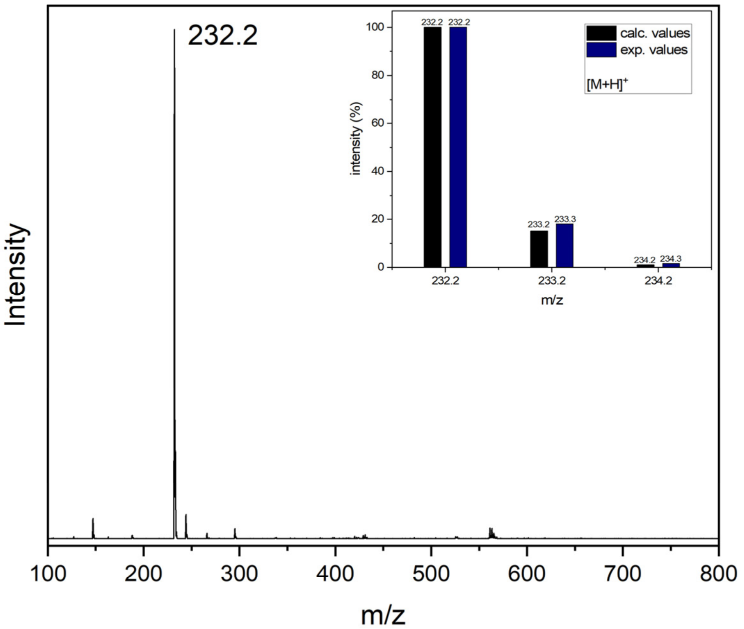

Figure S6. ESI-MS spectrum (positive mode) of 3,3-dimethyl-1-(1-methyl-1*H*-benzol[*d*]imidazole-2-yl)butane-2-amine (**L1**) with comparison of the experimental obtained data (blue) and calculated data (black).

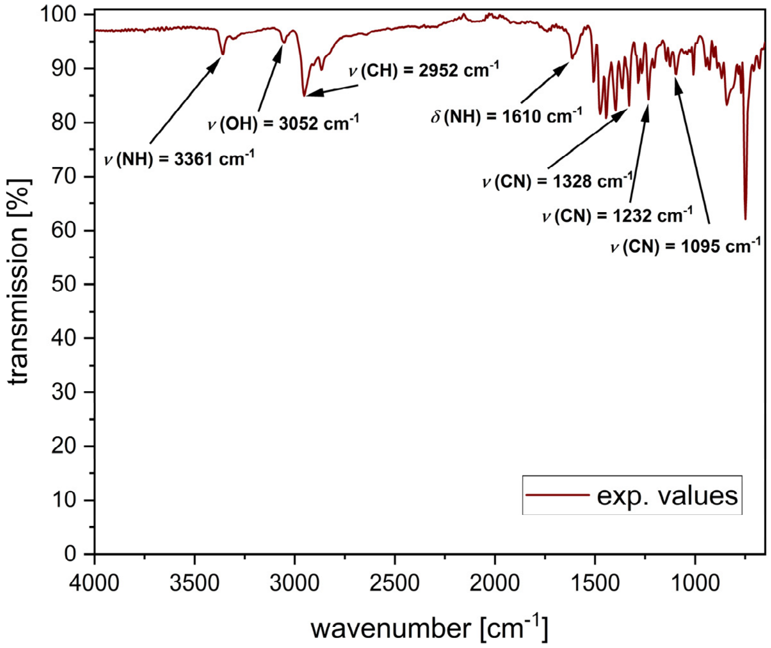

Figure S7. ATR-IR spectrum of 3,3-dimethyl-1-(1-methyl-1*H*-benzol[*d*]imidazole-2-yl)butane-2-amine (**L1**) at 20 °C.

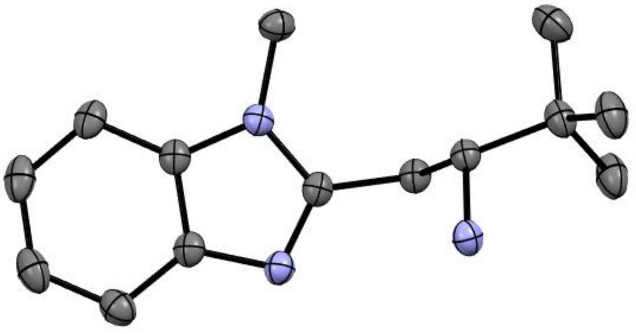

Figure S8. Molecular structure of **L1** obtained from sc-XRD measurements. The structure is shown in ORTEP representation with thermal ellipsoids at 50% probability level. H atoms are omitted for clarity. (Color code: N: blue, C: grey.)

Table S3. Crystallographic data of 3,3-dimethyl-1-(1-methyl-1*H*-benzol[*d*]imidazole-2-yl)butane-2-amine (**L1**) obtained from sc-XRD measurement.

Chemical sum formula	$C_{14}H_{21}N_3$
Chemical moiety formula	$C_{14}H_{21}N_3$
Molecular weight	231.41 g/mol
Temperature	100(2) K
Wavelength	0.71073 Å
Crystal system	triclinic
Space group	*P*-1
Unit cell parameters	a = 7.8808(5) Å
	b = 10.2764(7) Å
	c = 12.6928(8) Å
	α = 70.079(2) °
	β = 78.420(2) °
	γ = 71.236(2) °
Cell volume	910.26(10) Å3
Z	2
Crystal density	0.844
Absorption coefficient μ	0.051
F(000)	252
Crystal size	0.37 x 0.32 x 0.02 mm
Θ_{min} to θ_{max}	2.192 ° to 28.436 °
h, k, l range	$-10 \leq h \leq 10$; $-13 \leq k \leq 13$; $-16 \leq l \leq 16$
Total number of reflections (with I > 2σ(I))	4503
Number of parameters/restraints	166/0
R_1 factor	0.0598
wR_2 factor	0.1567
Goodness of Fit (GooF)	0.803
Deposition number	2411974

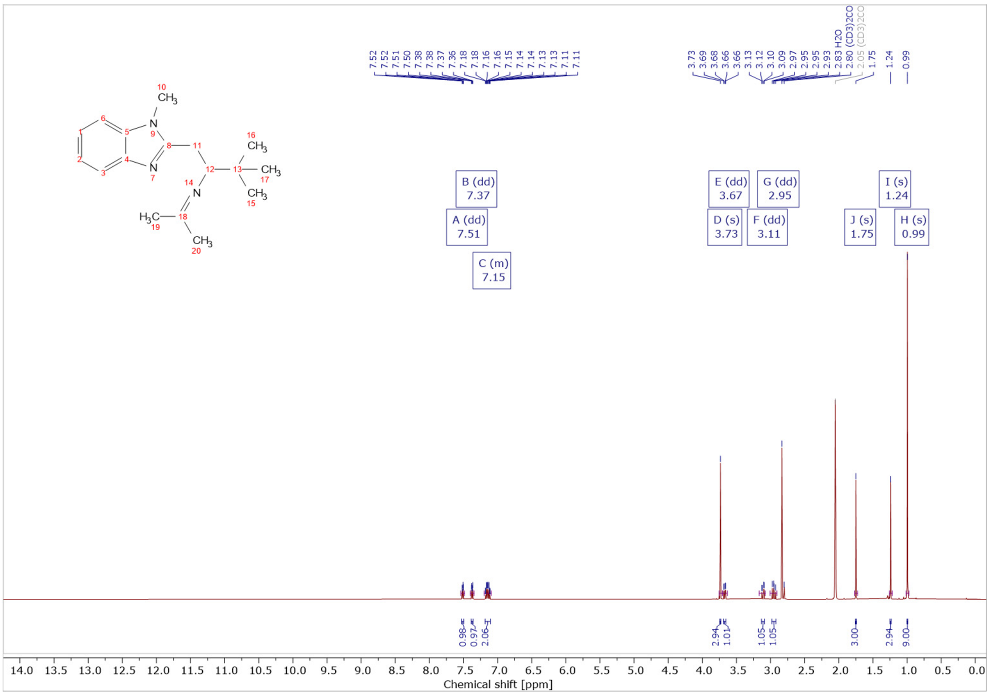

Figure S9. ^{1}H-NMR spectrum of 3,3-dimethyl-1-(1-methyl-1*H*-benzol[*d*]imidazole-2-yl)-*N*-(propane-2-ylidene)butane-2-amine (**L2**) in $(CD_3)_2CO$ at 20 °C.

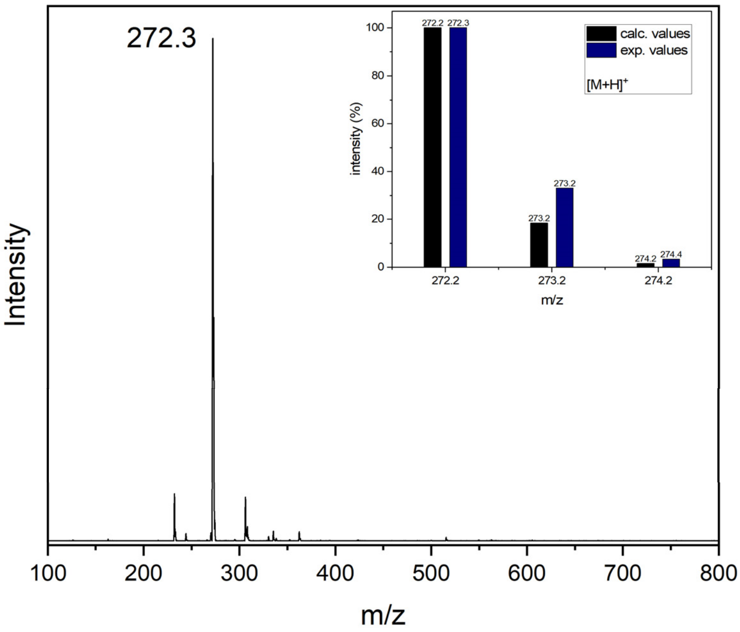

Figure S10. ESI-MS spectrum (positive mode) of 3,3-dimethyl-1-(1-methyl-1*H*-benzol[*d*]imidazole-2-yl)-*N*-(propan-2-ylidene)butane-2-amine (**L2**) with comparison of the experimental obtained data (blue) and calculated data (black).

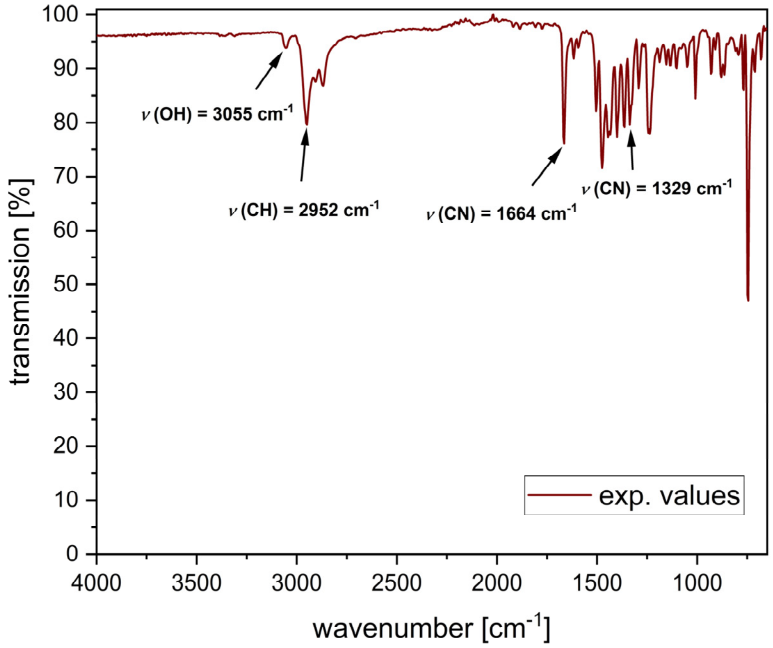

Figure S11. ATR-IR spectrum of 3,3-dimethyl-1-(1-methyl-1*H*-benzol[*d*]imidazole-2-yl)-*N*-(propane-2-ylidene)butane-2-amine (**L2**) at 20 °C.

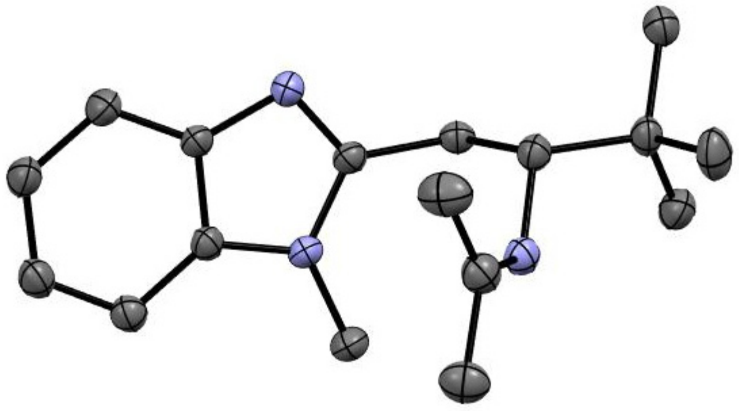

Figure S12. Molecular structure of **L2** obtained from sc-XRD measurements. The structure is shown in ORTEP representation with thermal ellipsoids at 50% probability level. H atoms are omitted for clarity. (Color code: N: blue, C: grey.)

Table S4. Crystallographic data of 3,3-dimethyl-1-(1-methyl-1*H*-benzol[*d*]imidazole-2-yl)-*N*-(propane-2-ylidene)butane-2-amine (**L2**) obtained from sc-XRD measurements.

Chemical sum formula	$C_{17}H_{25}N_3$
Chemical moiety formula	$C_{17}H_{25}N_3$
Molecular weight	271.40
Temperature	100(2) K
Wavelength	0.71073 Å
Crystal system	triclinic
Space group	*P*-1
Unit cell parameters	a = 6.5077(15) Å
	b = 11.049(2) Å
	c = 11.9351(19) Å
	α = 90.372(7) °
	β = 97.851(7) °
	γ = 106.793(7) °
Cell volume	812.9(3) $Å^3$
Z	2
Crystal density	1.109
Absorption coefficient μ	0.066
F(000)	296
Crystal size	0.49 x 0.19 x 0.09 mm
Θ_{min} to θ_{max}	2.524 ° to 28.431 °
h, k, l range	-8 ≤ h ≤ 8; -14 ≤ k ≤ 14; -13 ≤ l ≤ 15
Total number of reflections (with $I > 2\sigma(I)$)	4063
Number of parameters/restraints	187/0
R_1 factor	0.0674
wR_2 factor	0.1580
Goodness of Fit (GooF)	1.001
Deposition number	2411975

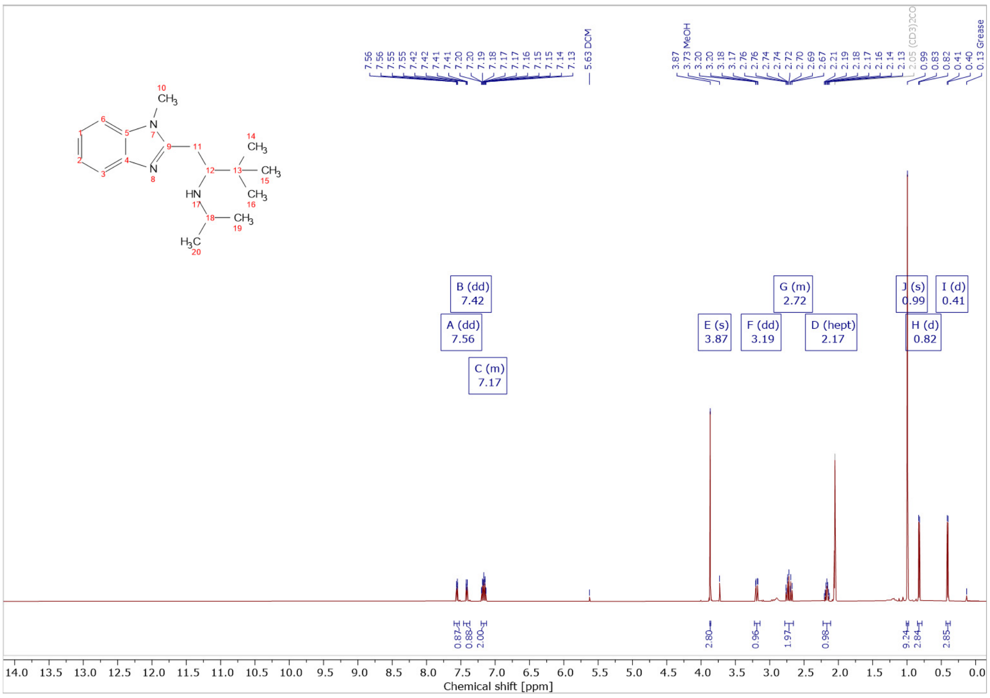

Figure S13. ^{1}H-NMR spectrum of *N*-isopropyl-3,3-dimethyl-1-(1-methyl-1*H*-benzol[*d*]imidazole-2-yl)butane-2-amine (**L3**) in $(CD_3)_2CO$ at 20 °C.

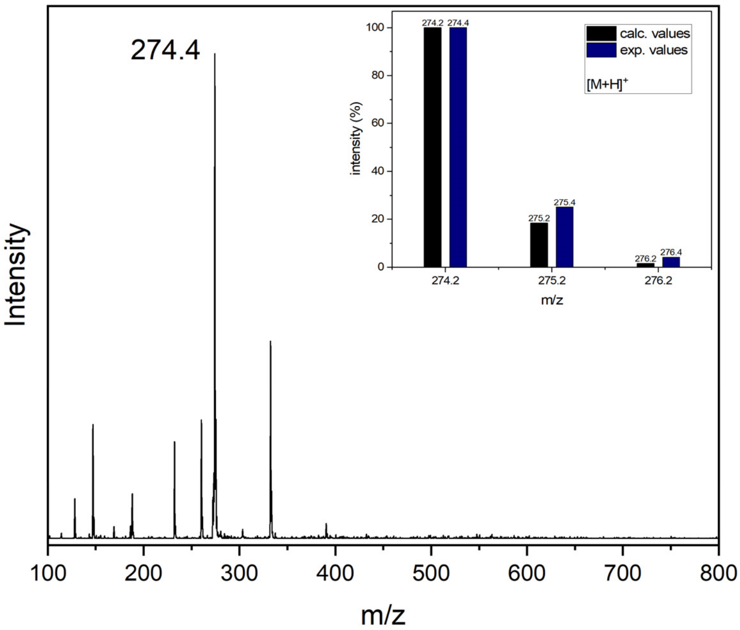

Figure S14. ESI-MS spectrum (positive mode) of *N*-isopropyl-3,3-dimethyl-1-(1-methyl-1*H*-benzol[*d*]imidazole-2-yl)butane-2-amine (**L3**) with comparison of the experimental obtained data (blue) and calculated data (black).

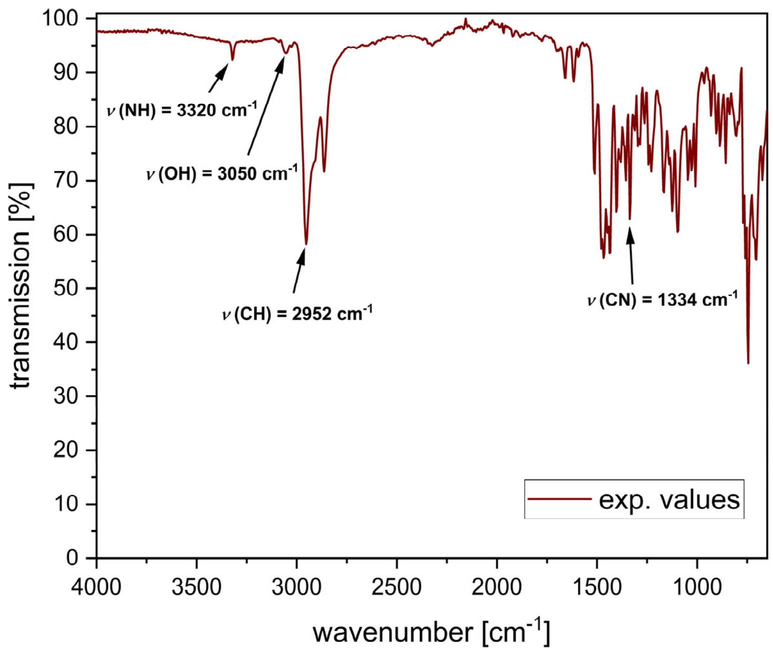

Figure S15. ATR-IR spectrum of *N*-isopropyl-3,3-dimethyl-1-(1-methyl-1*H*-benzol[*d*]imidazole-2-yl)butane-2-amine (**L3**) at 20 °C.

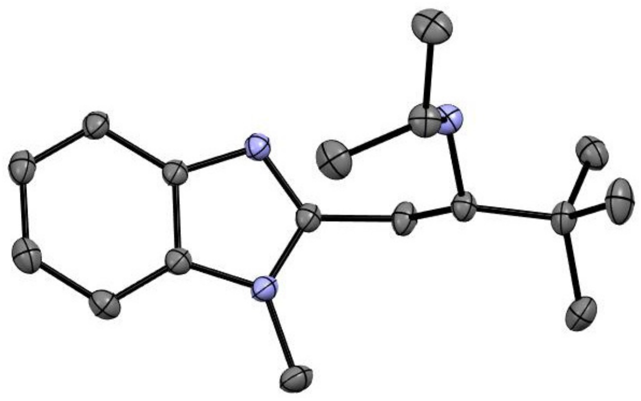

Figure S16. Molecular structure of **L3** obtained from sc-XRD measurements. The structure is shown in ORTEP representation with thermal ellipsoids at 50% probability level. H atoms are omitted for clarity. (Color code: N: blue, C: grey.)

Table S5. Crystallographic data of *N*-isopropyl-3,3-dimethyl-1-(1-methyl-1*H*-benzol[*d*]imidazole-2-yl)butane-2-amine (**L3**) obtained from sc-XRD measurements.

Chemical sum formula	$C_{17}H_{27}N_3$
Chemical moiety formula	$C_{17}H_{27}N_3$
Molecular weight	273.41 g/mol
Temperature	100(2) K
Wavelength	0.71073 Å
Crystal system	monoclinic
Space group	$P2_1/c$
Unit cell parameters	a = 10.9026(5) Å
	b = 16.1058(6) Å
	c = 9.6587(4) Å
	α = 90 °
	β = 106.605(2) °
	γ = 90 °
Cell volume	1625.29 Å^3
Z	4
Crystal density	1.117
Absorption coefficient μ	0.067
F(000)	600
Crystal size	0.53 x 0.29 x 0.21 mm
Θ_{min} to θ_{max}	2.33 ° to 24.07 °
h, k, l range	$-16 \leq h \leq 16$; $-24 \leq k \leq 24$; $-14 \leq l \leq 14$
Total number of reflections (with I > 2σ(I))	6203
Number of parameters/restraints	191/0
R_1 factor	0.0438
wR_2 factor	0.1212
Goodness of Fit (GooF)	1.003
Deposition number	2411976

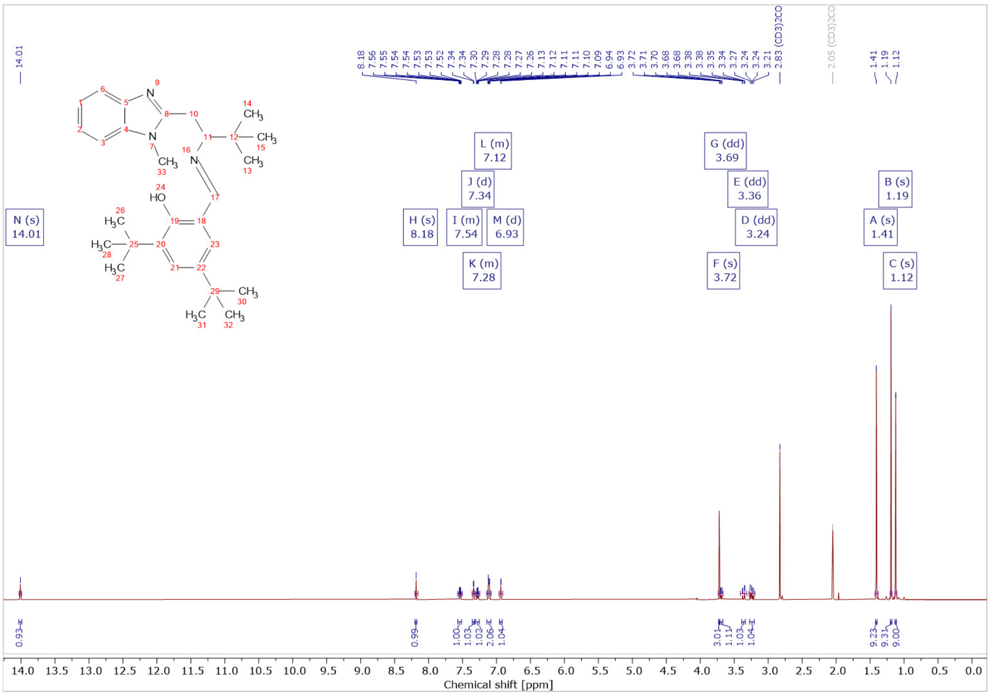

Figure S17. ^{1}H-NMR spectrum of (*Z*)-2,4-di-tert-butyl-6-(((3,3-dimethyl-1-(1-methyl-1*H*-benzol[*d*]imidazole-2-yl)butane-2-yl)imino)methyl)phenol (**L4**) in $(CD_3)_2CO$ at 20 °C.

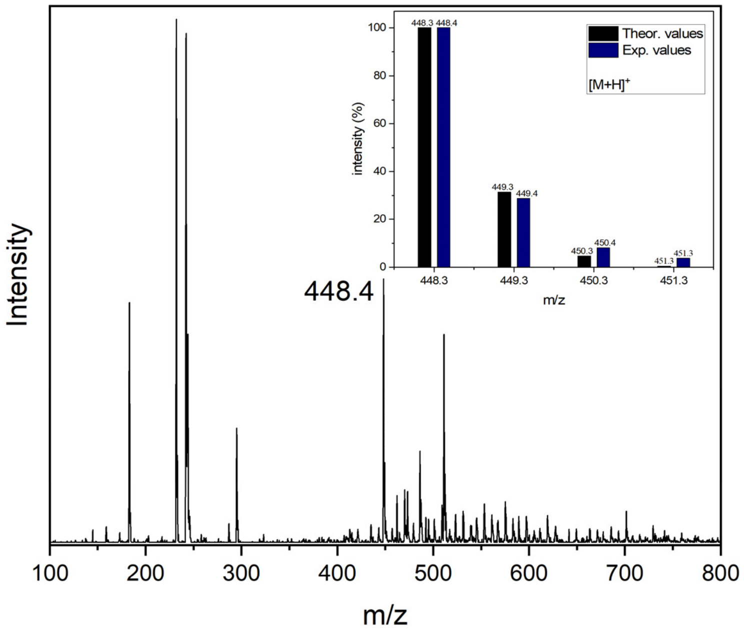

Figure S18. ESI-MS spectrum (positive mode) of (*Z*)-2,4-di-tert-butyl-6-(((3,3-dimethyl-1-(1-methyl-1*H*-benzol[*d*]imidazole-2-yl)butane-2-yl)imino)methyl)phenol (**L4**) with comparison of the experimental obtained data (blue) and calculated data (black).

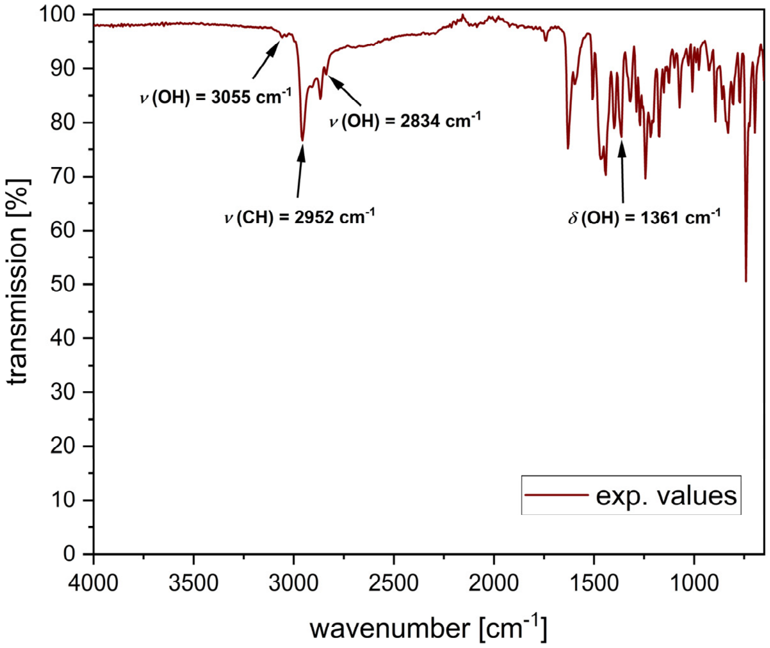

Figure S19. ATR-IR spectrum of (*Z*)-2,4-di-tert-butyl-6-(((3,3-dimethyl-1-(1-methyl-1*H*-benzol[*d*]imidazole-2-yl)butane-2-yl)imino)methyl)phenol (**L4**) at 20 °C.

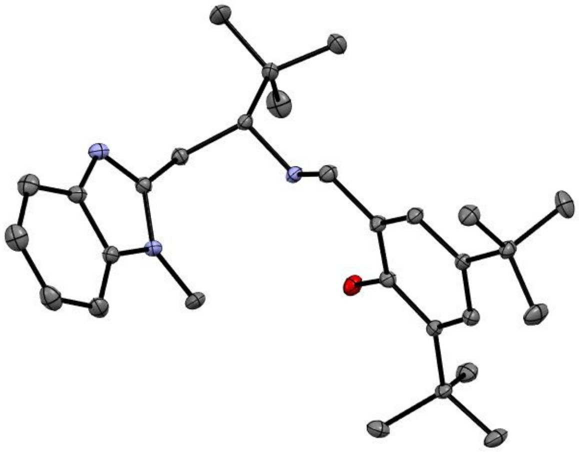

Figure S20. Molecular structure of **L4** obtained from sc-XRD measurements. The structure is shown in ORTEP representation with thermal ellipsoids at 50% probability level. H atoms are omitted for clarity. (Color code: N: blue, O: red, C: grey.)

Table S6. Crystallographic data of (*Z*)-2,4-di-tert-butyl-6-(((3,3-dimethyl-1-(1-methyl-1*H*-benzol[*d*]imidazole-2-yl)butane-2-yl)imino)methyl)phenol (**L4**) obtained from sc-XRD measurements.

Chemical sum formula	$C_{29}H_{41}N_3O$
Chemical moiety formula	$C_{29}H_{41}N_3O$
Molecular weight	447.65
Temperature	100(2) K
Wavelength	0.71073 Å
Crystal system	monoclinic
Space group	$P2_1/n$
Unit cell parameters	a = 10.2430(10) Å
	b = 10.3894(11) Å
	c = 25.225(2) Å
	α = 90 °
	β = 98.977(4) °
	γ = 90 °
Cell volume	2651.5(4) Å^3
Z	4
Crystal density	1.121
Absorption coefficient μ	0.068
F(000)	976
Crystal size	0.45 x 0.21 x 0.20 mm
Θ_{min} to θ_{max}	3.93 ° to 30.66 °
h, k, l range	-14 ≤ h ≤ 14; -14 ≤ k ≤ 14; -10 ≤ l ≤ 36
Total number of reflections (with I > 2σ(I))	8075
Number of parameters/restraints	313/0
R_1 factor	0.1052
wR_2 factor	0.3378
Goodness of Fit (GooF)	1.207
Deposition number	2411977

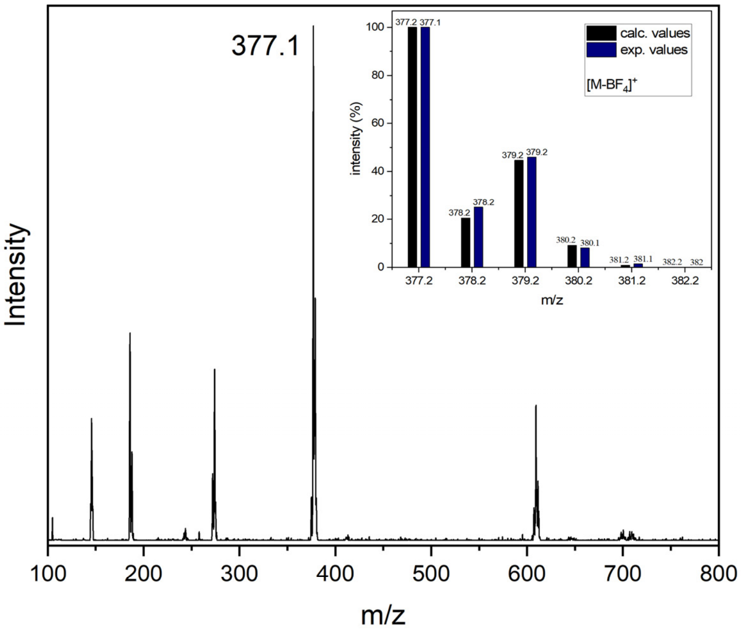

Figure S21. ESI-MS spectrum (positive mode) of Copper(I) [*N*-isopropyl-3,3-dimethyl-1-(1-methyl-1*H*-benzol[*d*]imidazole-2-yl)butane-2-amine] tetrafluoroborate (**Cu^{I}L3**) with comparison of the experimental obtained data (blue) and calculated data (black).

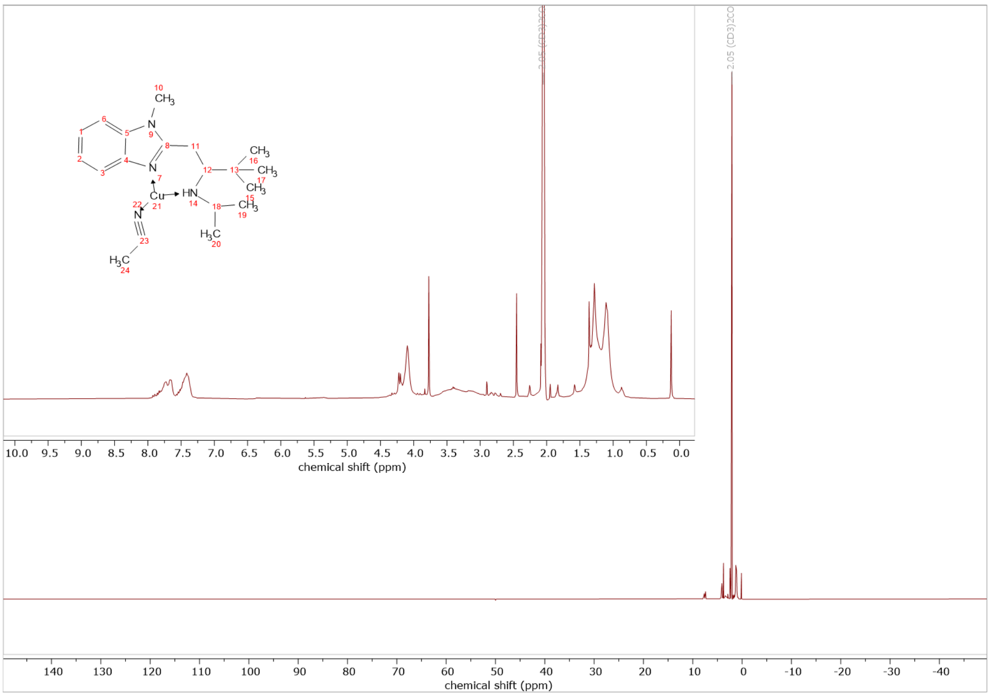

Figure S22. ^{1}H-NMR spectrum of Copper(I) [*N*-isopropyl-3,3-dimtyhl-1-(1-methyl-1*H*-benzol[*d*]imidazole-2-yl)butane-2-amine] tetrafluoroborate (**Cu^{I}L3**) in $(CD_3)_2CO$ at 20 °C.

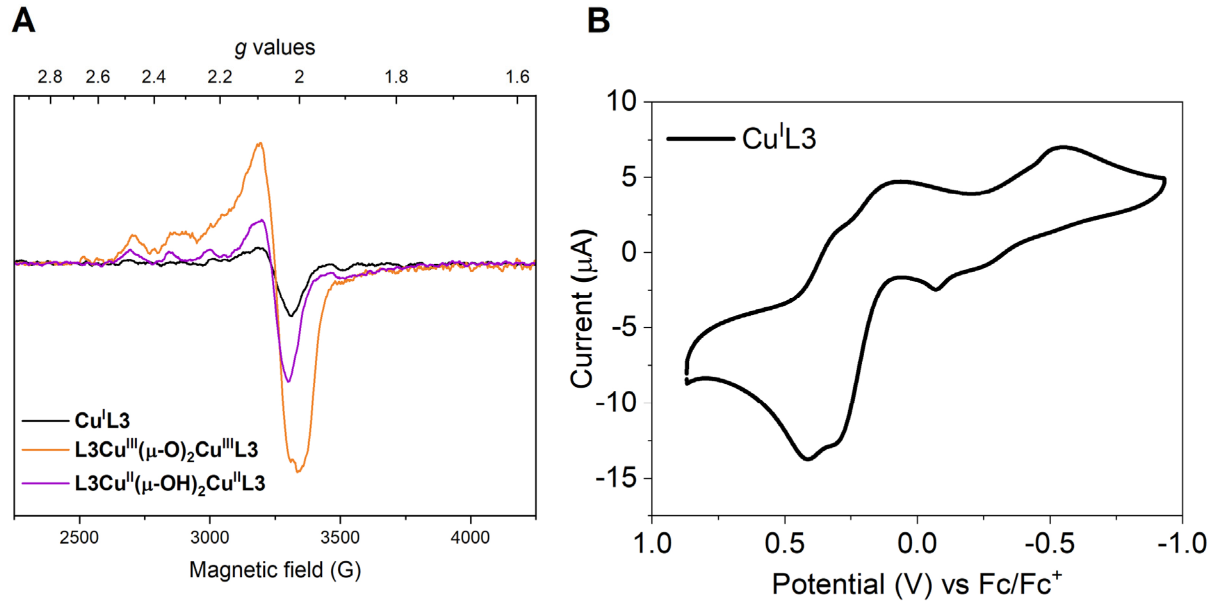

Figure S23. A) X-band EPR spectrum of **Cu^{I}L3**, **L3Cu^{III}(μ-O)$_2$Cu^{III}L3** and **L3Cu^{II}(μ-OH)$_2$Cu^{II}L3** in acetone (temperature: 13 K, modulation amplitude: 5 G, power: 0.02518 mW, 39 dB). Yield of EPR active species are 0.6%, 4.8% and 2.1%, respectively, compared to a Cu(II) standard; B) CV spectrum of **Cu^{I}L3**.

Table S7. Crystallographic data of Copper(I) [*N*-isopropyl-3,3-dimethyl-1-(1-methyl-1*H*-benzo[*d*]imidazole-2-yl)butane-2-amine] tetrafluoroborate (**Cu^IL3**) obtained from sc-XRD measurements.

Chemical sum formula	$C_{19}H_{30}BCuF_4N_4$
Chemical moiety formula	$C_{19}H_{30}CuN_4 \times BF_4$
Molecular weight	464.83
Temperature	100(2) K
Wavelength	0.71073 Å
Crystal system	triclinic
Space group	*P*-1
Unit cell parameters	*a* = 10.2945(11) Å
	b = 10.3423(11) Å
	c = 11.5853(14) Å
	α = 73.409(4) °
	β = 68.596(4) °
	γ = 74.447(4) °
Cell volume	1081.9(2) $Å^3$
Z	12
Crystal density	1.427
Absorption coefficient μ	1.055
F(000)	484
Crystal size	0.18 x 0.13 x 0.01 mm
Θ_{min} to θ_{max}	1.931 ° to 25.437 °
h, k, l range	-12 ≤ h ≤ 12; -12 ≤ k ≤ 12; -13 ≤ l ≤ 13
Total number of reflections (with I > 2σ(I))	3962
Number of parameters/restraints	330/0
R_1 factor	0.0302
wR_2 factor	0.0813
Goodness of Fit (GooF)	1.112
Deposition number	2388744

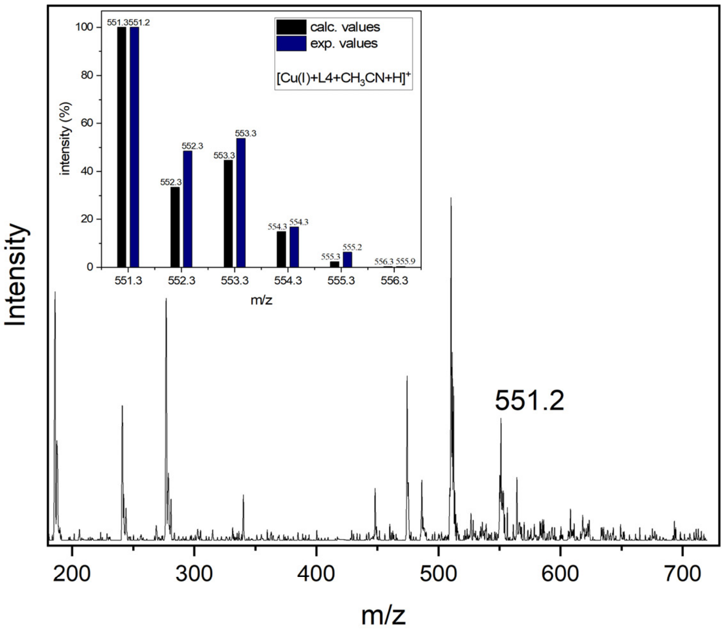

Figure S24. ESI-MS spectrum (positive mode) of dicopper(I) bis[(*Z*)-2,4-di-*tert*-butyl-6-(((3,3-dimethyl-1-(1-methyl-1*H*-benzol[*d*]imidazole-2-yl)butane-2-yl)imino)methyl)phenolate] (**Cu$^{I}_{2}$L4$_2$**) with comparison of the experimental obtained data (blue) and calculated data (black).

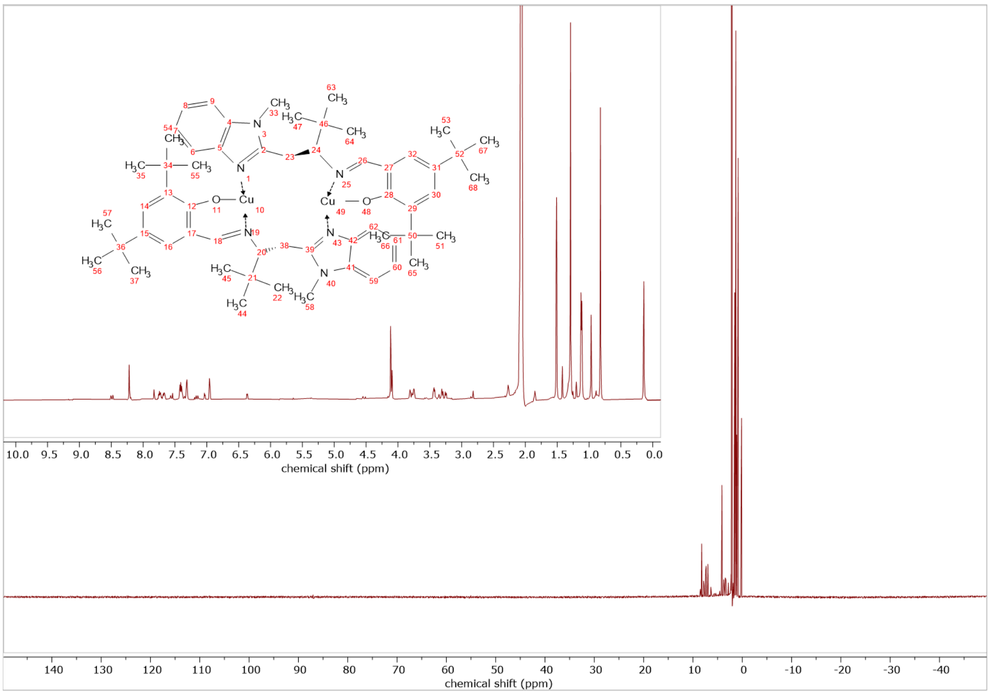

Figure S25. ^{1}H-NMR spectrum of dicopper(I) bis[(*Z*)-2,4-di-*tert*-butyl-6-(((3,3-dimethyl-1-(1-methyl-1*H*-benzol[*d*]imidazole-2-yl)butane-2-yl)imino)methyl)phenolate] (**Cu$^{I}_{2}$L4$_2$**) in $(CD_3)_2CO$ at 20 °C.

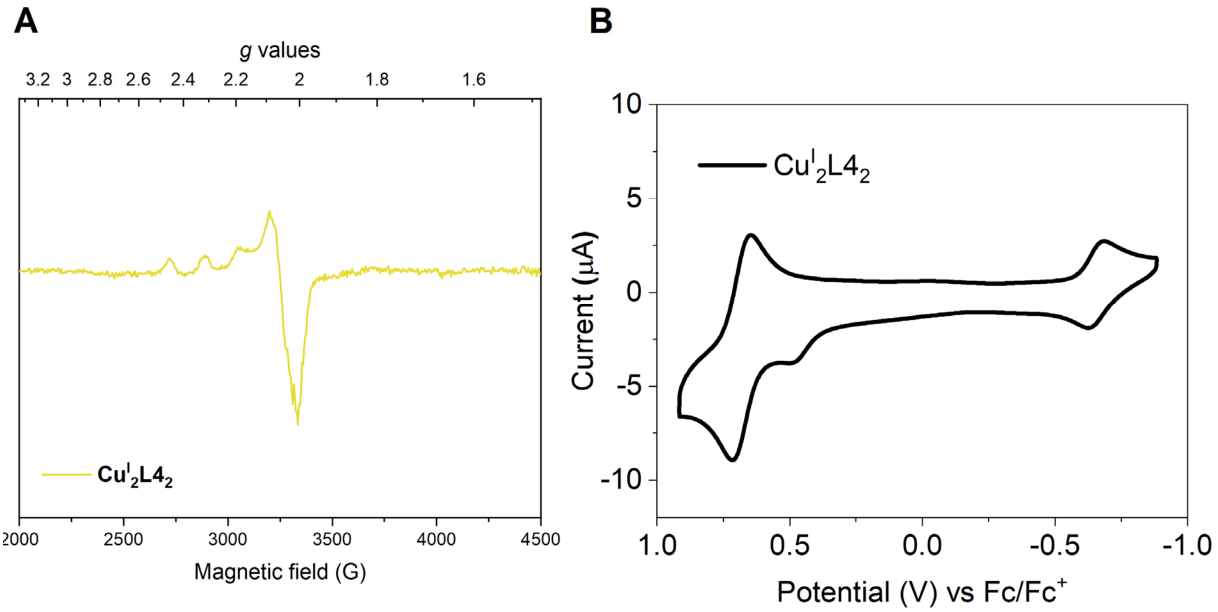

Figure S26. A) X-band EPR spectrum of **Cu$^I_2$L4$_2$** in MeCN (temperature: 13 K, modulation amplitude: 5 G, power: 0.02518 mW, 39 dB). Yield of EPR active species is 5.2% compared to a Cu(II) standard; B) CV spectrum of **Cu$^I_2$L4$_2$**.

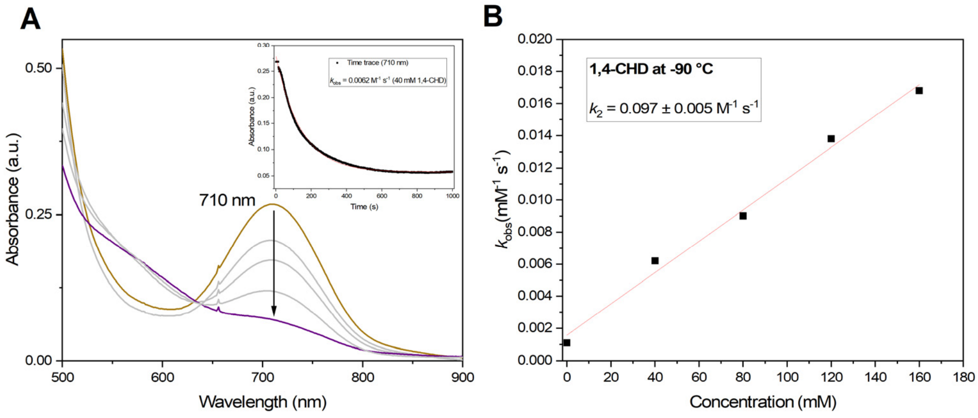

Figure S27. A) UV/Vis spectrum of the reaction between **L3Cu^{III}(μ-O)$_2$Cu^{III}L3** and 20 eq. 1,4-CHD at -90 °C in acetone; B) k_{obs} vs. [1,4-CHD] plot for k_2 determination of the reaction between **L3Cu^{III}(μ-O)$_2$Cu^{III}L3** and selected equivalents of 1,4-CHD at -90 °C in acetone ([**Cu^{I}L3**] = 2 mM). k_{obs} values were determined by pseudo-first order fit of the decay of the 710 nm band.

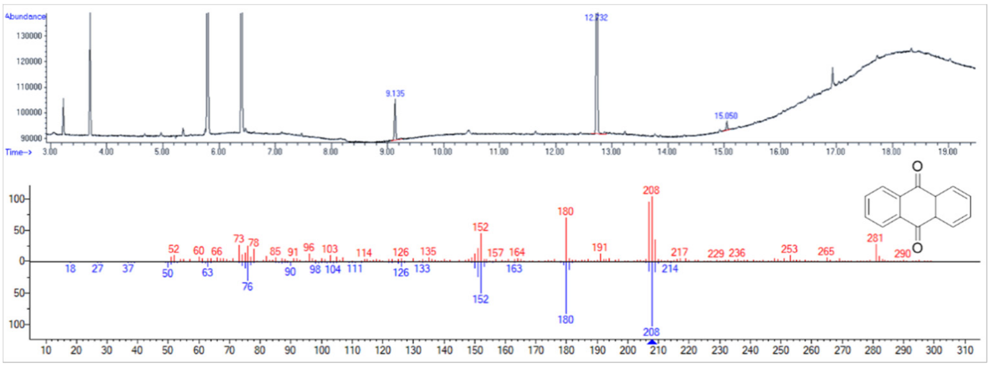

Figure S28. GC-FID spectrum of the reaction mixture of **L3Cu^{III}(µ-O)$_2$Cu^{III}L3** and DHA with the corresponding experimental and calculated mass spectrum of the product. Retention time (s): biphenyl: 9.135, DHA: 12.732, anthraquinone: 15.050.

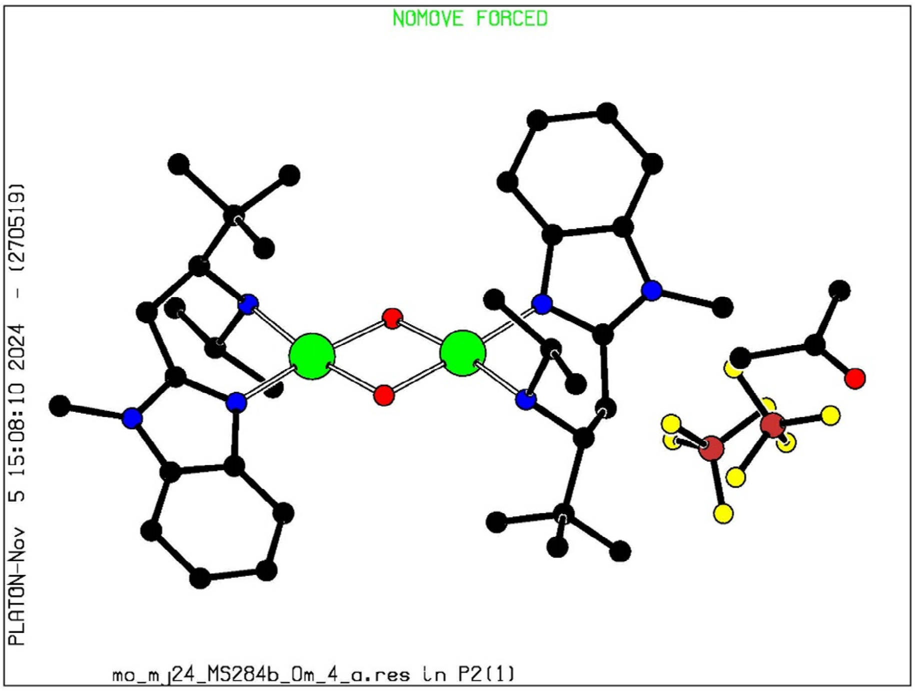

Figure S29. Molecular structure of **L3Cu^{II}(µ-OH)$_2$Cu^{II}L3** obtained from sc-XRD measurements. Due to low quality data we were unable to discuss further bond distances and angles but only to determine the connectivity.

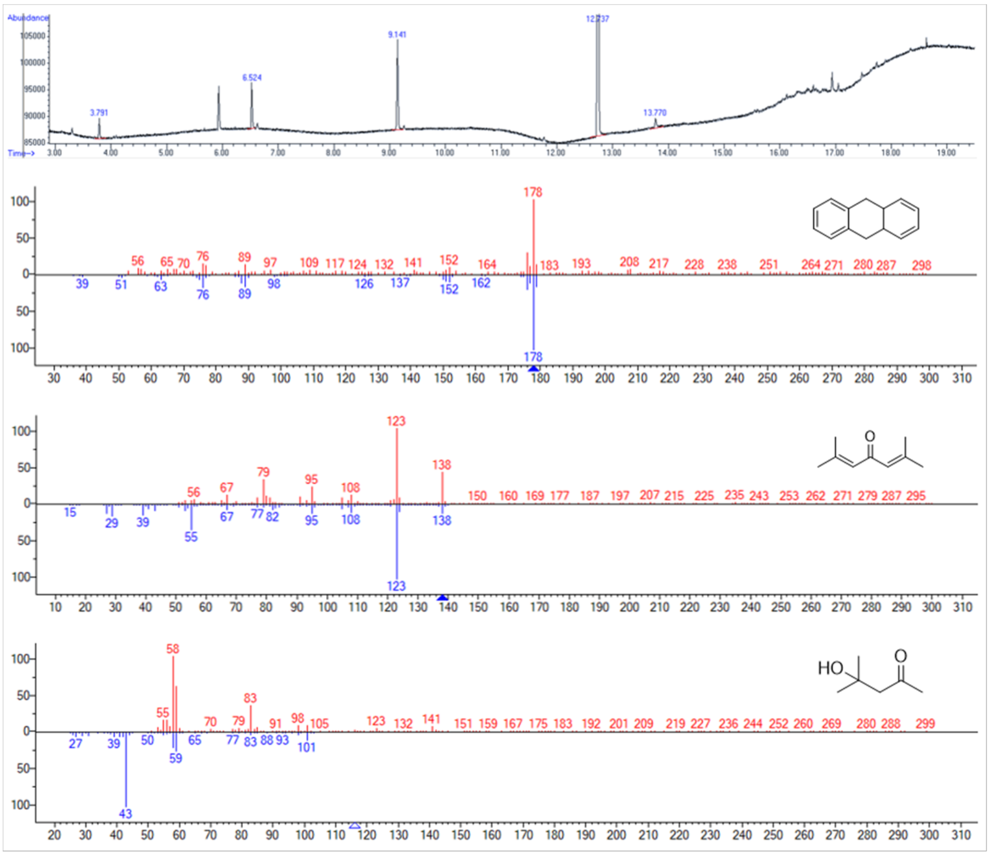

Figure S30. GC-FID spectrum of the reaction mixture of **L3Cu^{II}(μ-OH)$_2$Cu^{II}L3** and DHA with the corresponding experimental and calculated mass spectra of the products. Retention time (s): phorone: 3.791, 4-hydroxy-4-methylpentane-2-one: 6.524, biphenyl: 9.141, DHA: 12.737, anthracene: 13.770.

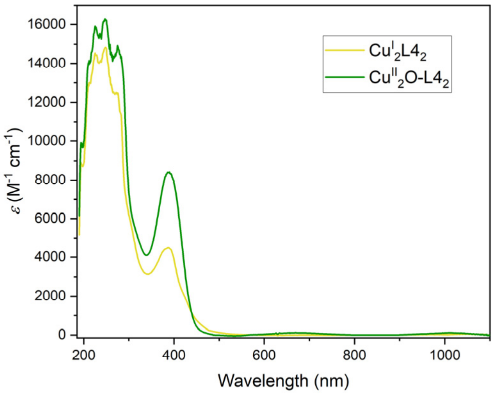

Figure S31. UV/Vis spectrum of **Cu$^{I}_2$L4$_2$** and **Cu$^{II}_2$O-L4$_2$** in MeCN at 20 °C (conc. 1 mM).

Table S8. Crystallographic data of Dicopper(I) bis[(*Z*)-2,4-di-*tert*-butyl-6-(((3,3-dimethyl-1-(1-methyl-1*H*-benzol[*d*]imidazole-2-yl)butane-2-yl)imino)methyl)phenolate] (**$Cu^{I}_{2}L4_{2}$**) obtained from sc-XRD measurements.

Chemical sum formula	$C_{58}H_{80}Cu_2N_6O_2$
Chemical moiety formula	$C_{58}H_{80}Cu_2N_6O_2$
Molecular weight	1020.36
Temperature	100(2) K
Wavelength	0.71073 Å
Crystal system	monoclinic
Space group	$P2_1/n$
Unit cell parameters	a = 16.6543(6) Å
	b = 11.9303(5) Å
	c = 27.4410(12) Å
	α = 90 °
	β = 95.735(2) °
	γ = 90 °
Cell volume	5425.0(4) Å^3
Z	4
Crystal density	1.244
Absorption coefficient μ	0.830
F(000)	2168
Crystal size	0.17 x 0.14 x 0.08 mm
Θ_{min} to θ_{max}	2.267 ° to 28.351 °
h, k, l range	-20 ≤ h ≤ 22; -15 ≤ k ≤ 15; -36 ≤ l ≤ 36
Total number of reflections (with I > 2σ(I))	13508
Number of parameters/restraints	673/100
R_1 factor	0.0322
wR_2 factor	0.0825
Goodness of Fit (GooF)	1.026
Deposition number	2411978

Table S9. Crystallographic data of **Cu^{II}_2O-L4$_2$ × 4 C_2H_3N** and **Cu^{II}_2O-L4$_2$ × 2 C_2H_3N × 2 C_7H_8O** obtained from sc-XRD measurements.

Chemical sum formula	$C_{124}H_{168}Cu_4N_{16}O_8$	$C_{134}H_{178}Cu_4N_{14}O_{10}$
Chemical moiety formula	$(C_{58}H_{78}Cu_2N_6O_4 \times 2\ C_2H_3N)_2$	$(C_{58}H_{78}Cu_2N_6O_4 \times C_2H_3N \times C_7H_8O)_2$
Molecular weight	2265.00	2399.17
Temperature	100(2) K	100(2) K
Wavelength	0.71073 Å	0.71073 Å
Crystal system	monoclinic	triclinic
Space group	$P2_1/c$	P-1
Unit cell parameters	a = 23.634(4) Å	a = 10.6165(16) Å
	b = 10.6113(16) Å	b = 16.205(2) Å
	c = 28.345(4) Å	c = 23.384(3) Å
	α = 90 °	α = 71.630(5) °
	β = 110.894(5) °	β = 78.089(5) °
	γ = 90 °	γ = 72.104(5) °
Cell volume	6641.1(18) Å^3	3624.5(9) Å^3
Z	4	2
Crystal density	1.215	1.236
Absorption coefficient μ	0.693	0.643
F(000)	2584	1436
Crystal size	0.44 x 0.05 x 0.03 mm	0.29 x 0.04 x 0.04 mm
Θ_{min} to θ_{max}	2.056 ° to 25.458 °	2.33 ° to 22.19 °
h, k, l range	-28 ≤ h ≤ 28; -12 ≤ k ≤ 12; -34 ≤ l ≤ 32	-13 ≤ h ≤ 13; -20 ≤ k ≤ 20; -29 ≤ l ≤ 29
Total number of reflections (with I > 2σ(I))	12234	14939
Number of parameters/restraints	703/0	874/1363
R_1 factor	0.1182	0.0711
wR_2 factor	0.2828	0.1623
Goodness of Fit (GooF)	1.090	0.985
Deposition number	2411979	2411980

Table S10. Product yields (%) of the aerobic alcohol oxidation reactions using either **$Cu^{I}L3$** in acetone or **$Cu^{I}_{2}L4_{2}$** in acetonitrile together with TEMPO (and NMI) under otherwise identical reaction conditions.

Substrate	Product	$Cu^{I}L3$	$Cu^{I}_{2}L4_{2}$	$Cu^{I}_{2}L2_{2}$[6]
Benzyl alcohol	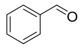	quant. (69)[a]	10	quant. (20)[a]
Hexanol	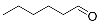	18 (21)[a]	1	37 (2)[a]
2-Ethoxyethanol	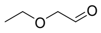	9	8	16
2-hydroxymethylpiperidine	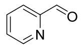	8	6	9

[a] obtained yields in absence of NMI.

Table S11. Solvent-dependent study aerobic alcohol oxidation reactions using **$Cu^{I}L3$** and **$Cu^{I}_{2}L4_{2}$** in acetone or acetonitrile together with TEMPO and NMI. Yields are given in %.

Substrate	Product	$Cu^{I}L3$ (acetone)	$Cu^{I}L3$ (MeCN)	$Cu^{I}_{2}L4_{2}$ (acetone)	$Cu^{I}_{2}L4_{2}$ (MeCN)
Benzyl alcohol	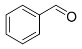	quant.	25	1	10
Hexanol	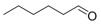	18	6	0	1

Table S12. Temperature-dependent study on the aerobic oxidation of hexanol using **$Cu^{I}L3$** in acetone together with TEMPO and NMI. Yields are given in %.

Substrate	Product	0 °C	20 °C	40 °C
Hexanol	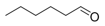	10	18	9
